# Supplementary material for: Chromatin activity identifies differential gene regulation across human ancestries
Source: Genome Biol. 2024 Jan 15;25:21. doi: 10.1186/s13059-024-03165-2 (PMC10789071; doi:10.1186/s13059-024-03165-2)
Supplement: Supplementary file 1 — Additional file 1. [file 13059_2024_3165_MOESM1_ESM.docx]

**Supplemental text**

**Differential CRE activity is linked to differential expression between ancestries**

*Differential activity-by-contact scores*

Of the 50,478 CRE-target gene (enhancer-gene) pairs with nonzero ATAC and HiChIP signal in all samples and passing enhancer-gene pair candidacy thresholds (see Methods) in at least one sample, 2,911 (~5.8%) had differential ABC scores (diff-ABC) between AFR and EUR populations (diff-ABC P < 0.05, see Methods). Of these diff-ABC enhancer-gene pairs, 1,418 had higher ABC scores in AFR (AFR high ABC) and 1,493 had higher ABC in EUR (EUR high ABC). These AFR and EUR high ABC enhancer-gene pairs comprised 1,199 and 1,291 distinct target genes, and 1,122 and 1,184 distinct CREs, respectively.

We found our diff-ABC CRE target genes were enriched for ancestry DE in just five of the 22 contexts assayed across these studies (Fisher’s P = 2.10 x 10^-3^ - 6.79 x 10^-7^, Bonferroni-corrected P-value threshold = 2.27 x 10^-3^, see Methods; Additional file 1: Fig. S2), none of which were the ABC-assayed context. These results are consistent with the inclusion of contact frequency in ABC scores diluting the association of activity levels with differential expression.

*Outlier removal*

We observed that both CEU replicates were significant outliers in ChIP scores and to a lesser extent ATAC scores in PCAs and heatmaps of enhancer-gene pair scores (Additional file 1: Fig. S4-7). CEU LCLs were the first established of any of the 1000 Genomes populations and their age has been proposed to drive their outlier status in previous studies [1]. Thus, to be conservative and ensure any subsequent results would not be driven solely by CEU, we removed this population, redefined enhancer-gene pairs, and computed scores for downstream analyses using t­he remaining 14 samples.

*iHS analysis*

To assess evidence for selection with statistics beyond *F_ST_*, we performed tests analogous to those in Fig. 4a using iHS scores from Johnson and Voight (2018) [2] and observe no significant differences (Additional file 1: Fig. S19, see Methods); however, we do not expect signatures of within population selection such as iHS to necessarily be enriched in CREs that are differentially active between ancestries. Moreover, since iHS detects long haplotypes that have quickly risen in frequency, it lacks the SNP-level resolution required for the CRE-level tests in this study.

**Supplemental figures**

**
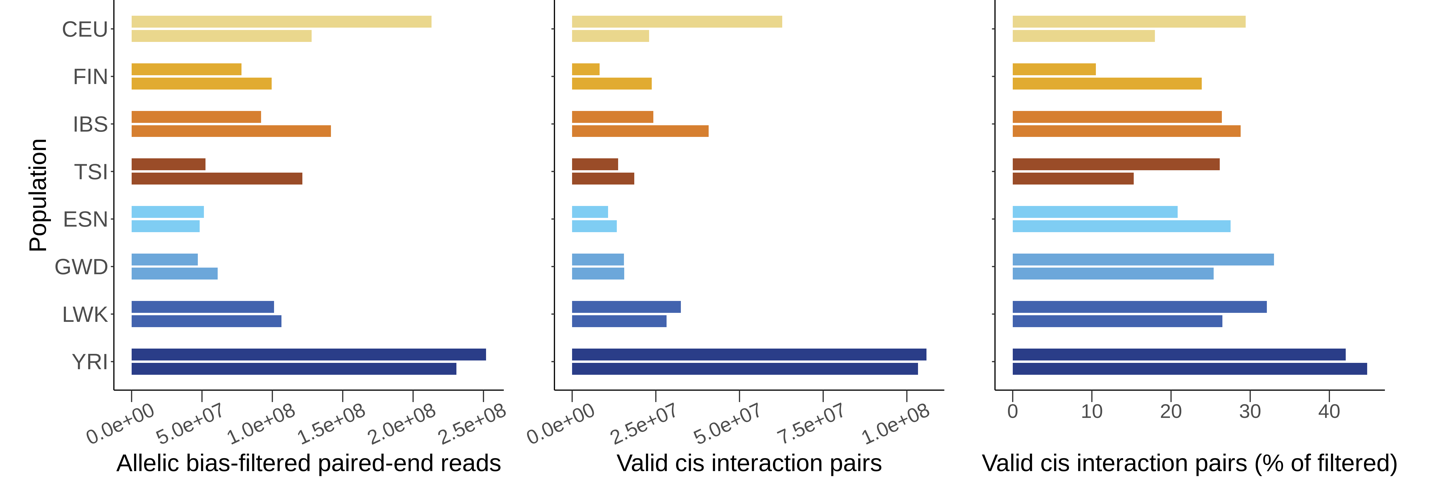
Figure S1. HiChIP reads filtering for use in ABC scores.** Top bars are replicate 1 and bottom bars are replicate 2 for each population. Contact maps were generated from valid *cis* interaction pairs at 5 Kb resolution for computing ABC, ChIP, and HiC scores.


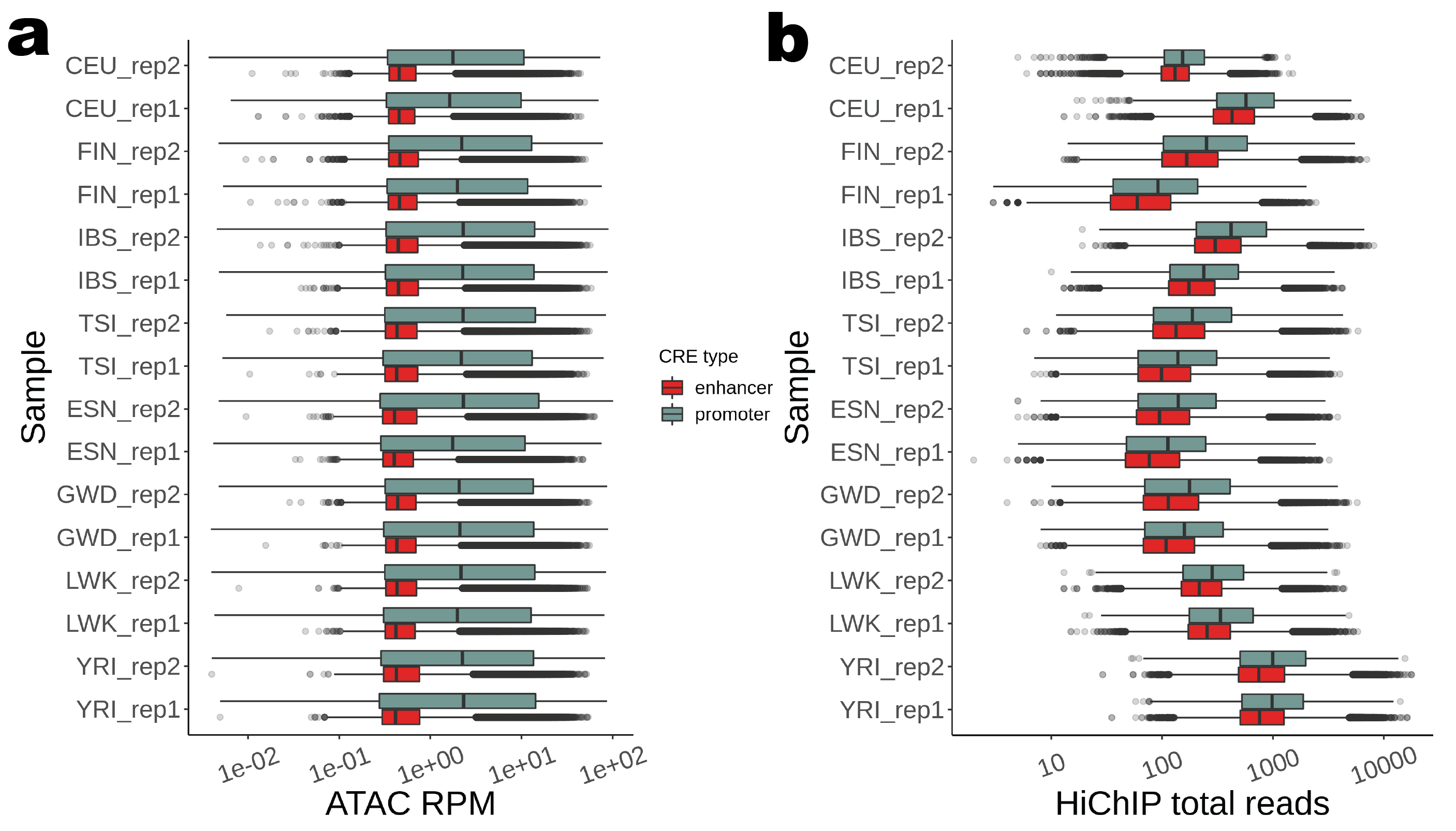


**Figure S2. ATAC and HiChIP coverage in enhancers and promoters.** Number of reads in each CRE shown as boxplots grouped by CRE type before quantile normalization. **a)** ATAC-seq reads per million sequenced reads (RPM). **b)** HiChIP total reads, where one read can contribute to multiple CREs (see Methods).

**
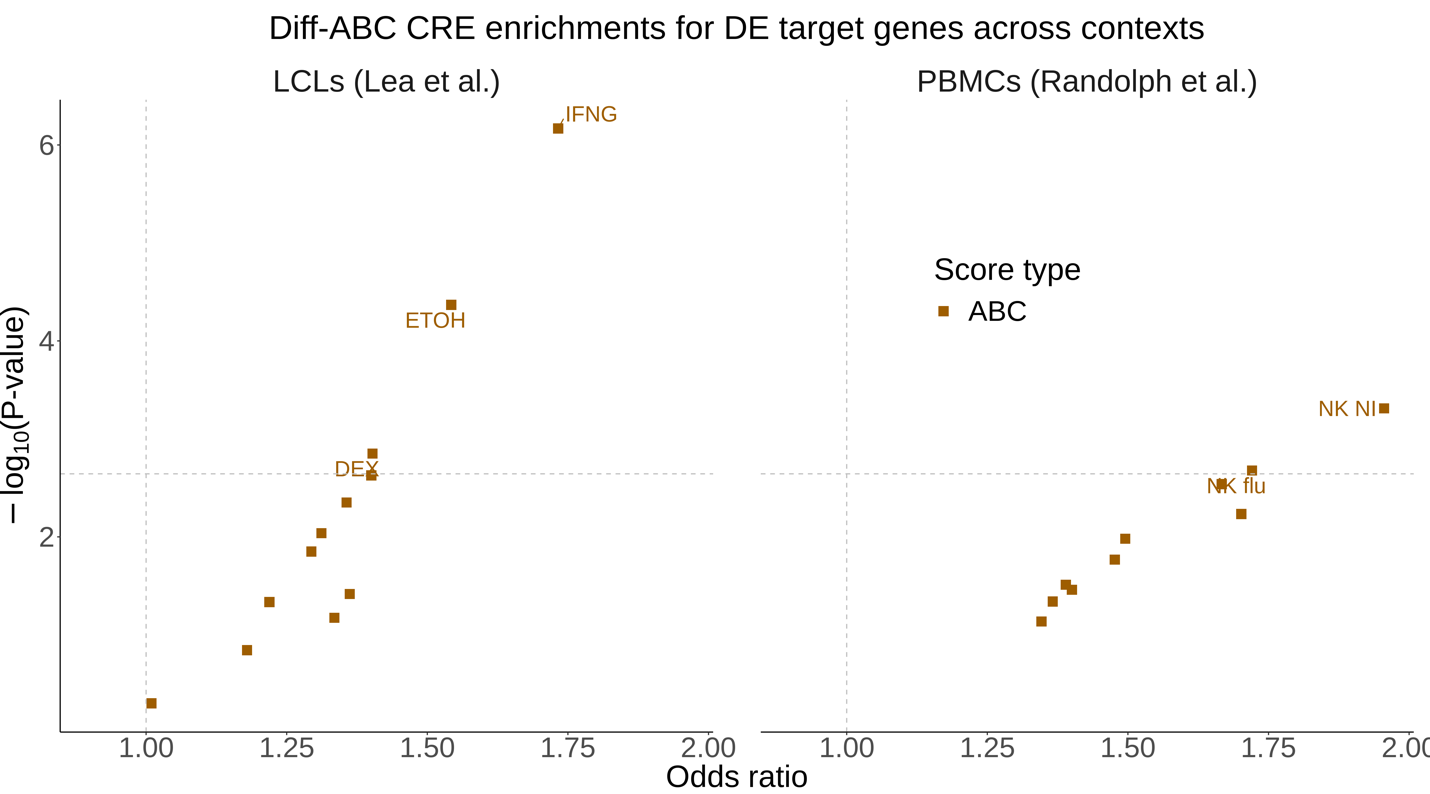
**

**Figure S3. Diff-ABC (CEU-inclusive) enrichments for DE target genes across conditions and cell types.** See Fig. 2a,b legend.

**
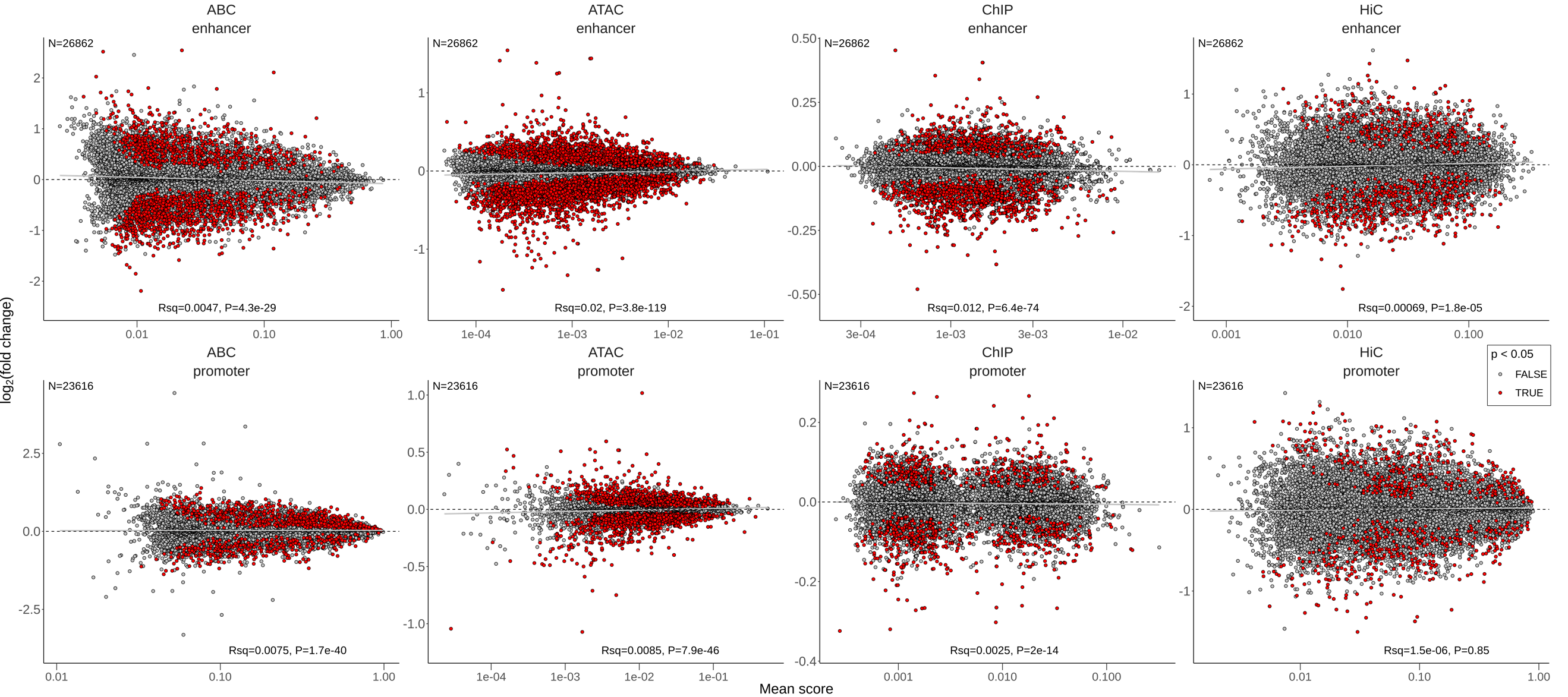
**

**Figure S4. MA-style plots of diff-CRE (CEU-inclusive) directionality.** The mean score (“A” scale) is plotted against the log_2_ ratio (“M” scale) or fold change of mean EUR score over mean AFR score for each CRE and score type. Red points are E-G pairs with diff-score P < 0.05.


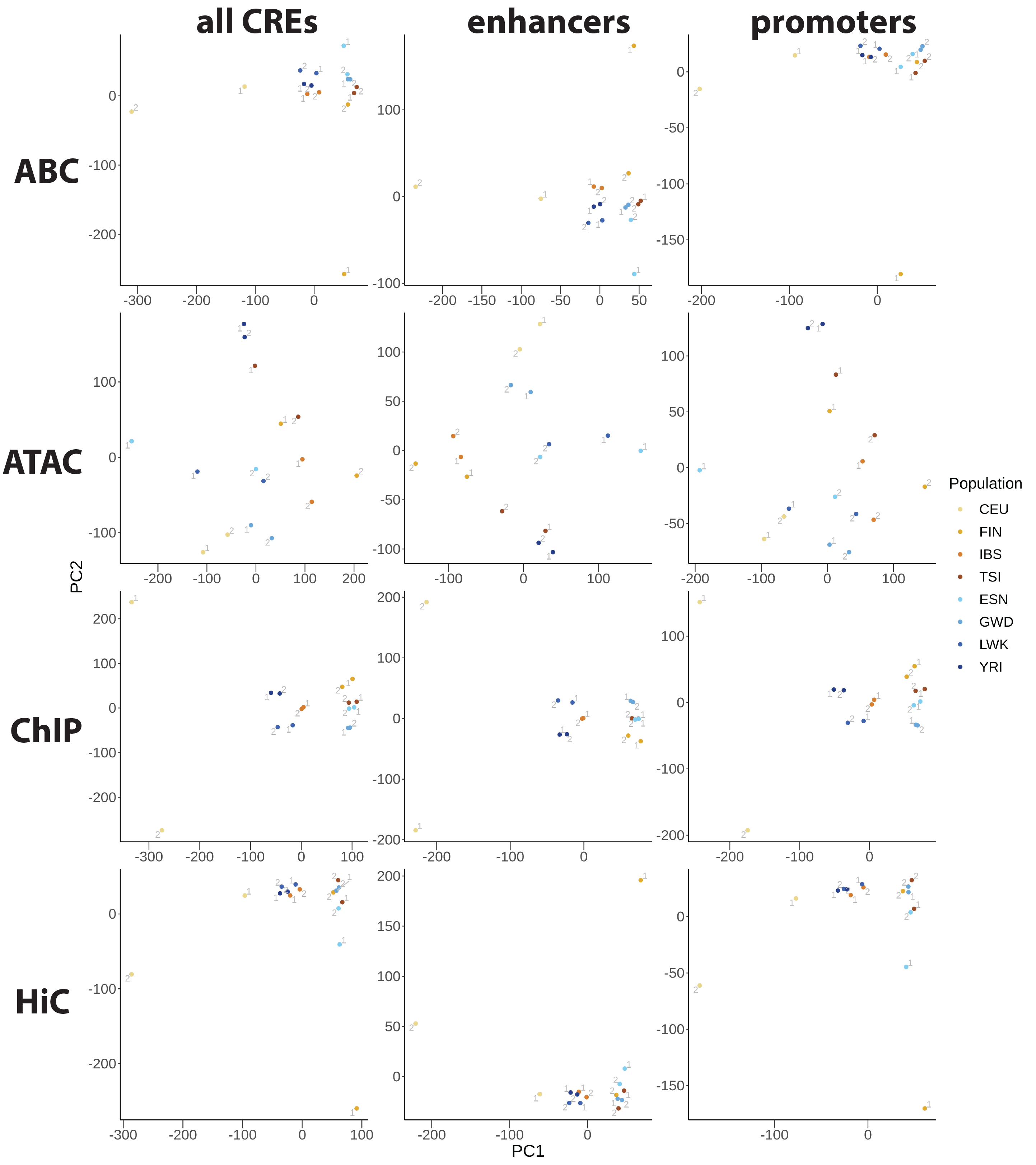


**Figure S5. PCAs of all E-G pair scores (CEU-inclusive).** Replicate numbers are displayed next to each point.


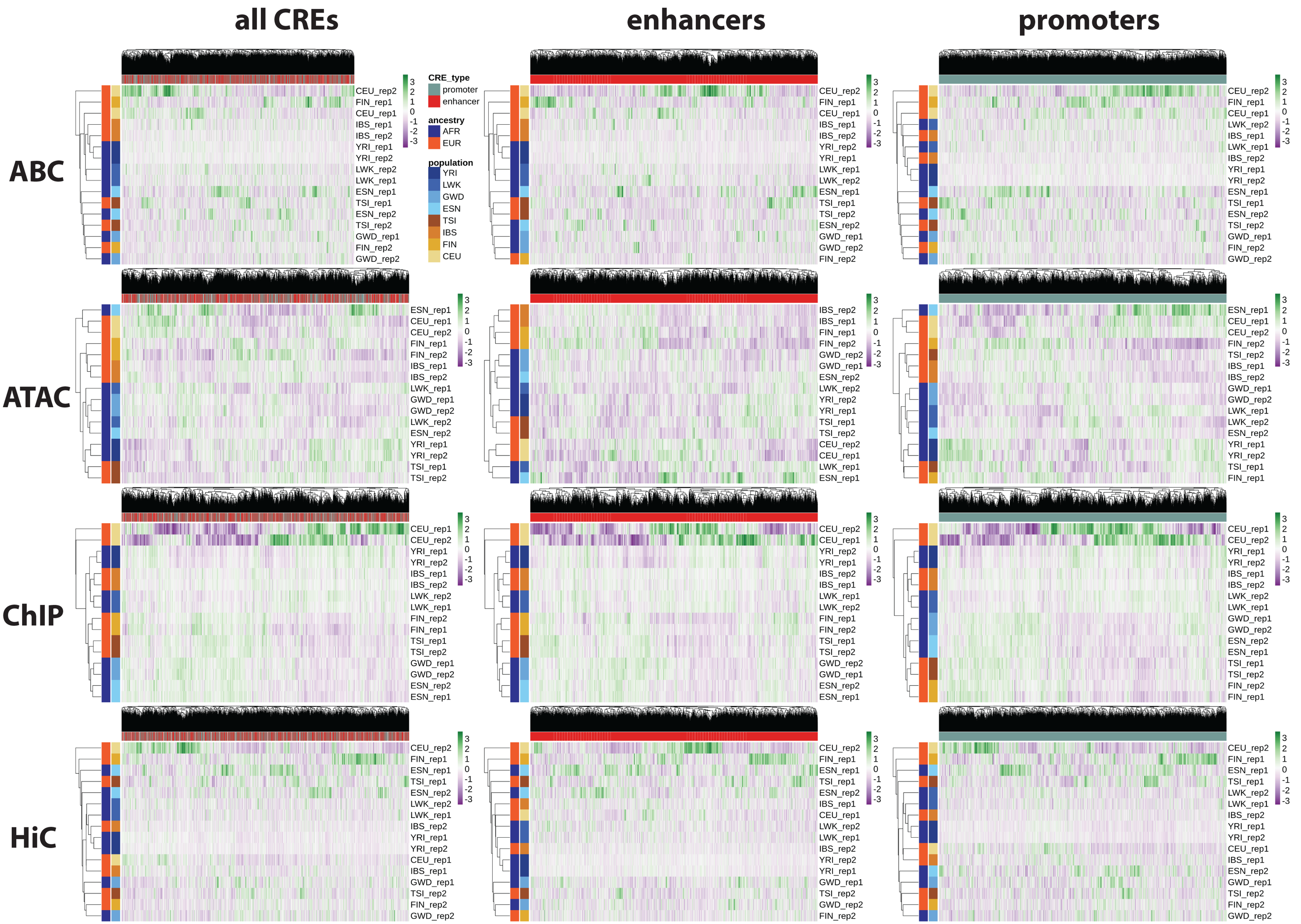


**Figure S6. Heatmaps of all E-G pair scores (CEU-inclusive) with hierarchical clustering.**

**
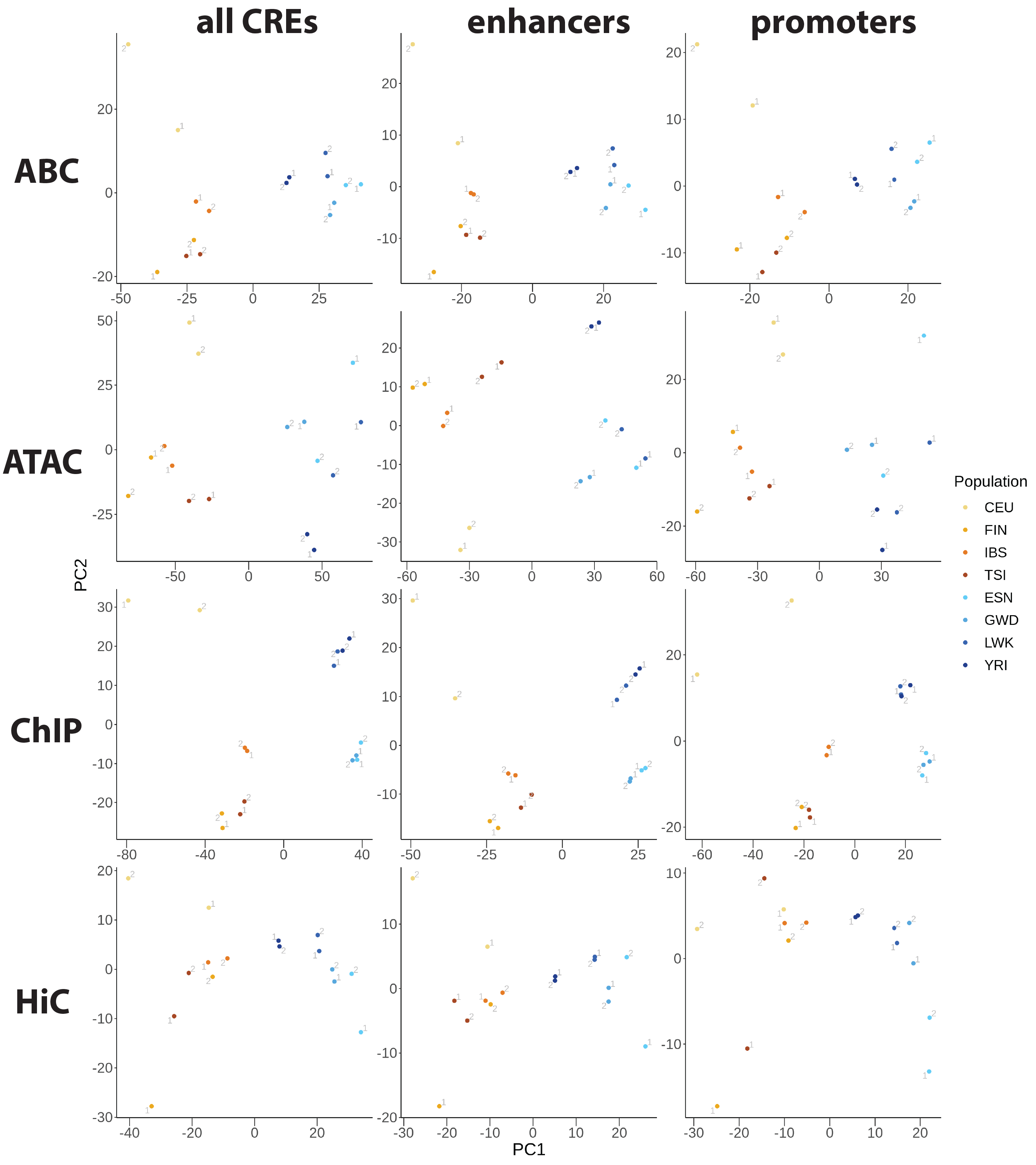
**

**Figure S7. PCAs of top diff-E-G pair scores (CEU-inclusive).** E-G pairs were subset first by diff-score P < 0.05, then by lowest P-value per gene, then by CRE type indicated by column names before performing PCA on each score type (row names). Replicate numbers are displayed next to each point.

**
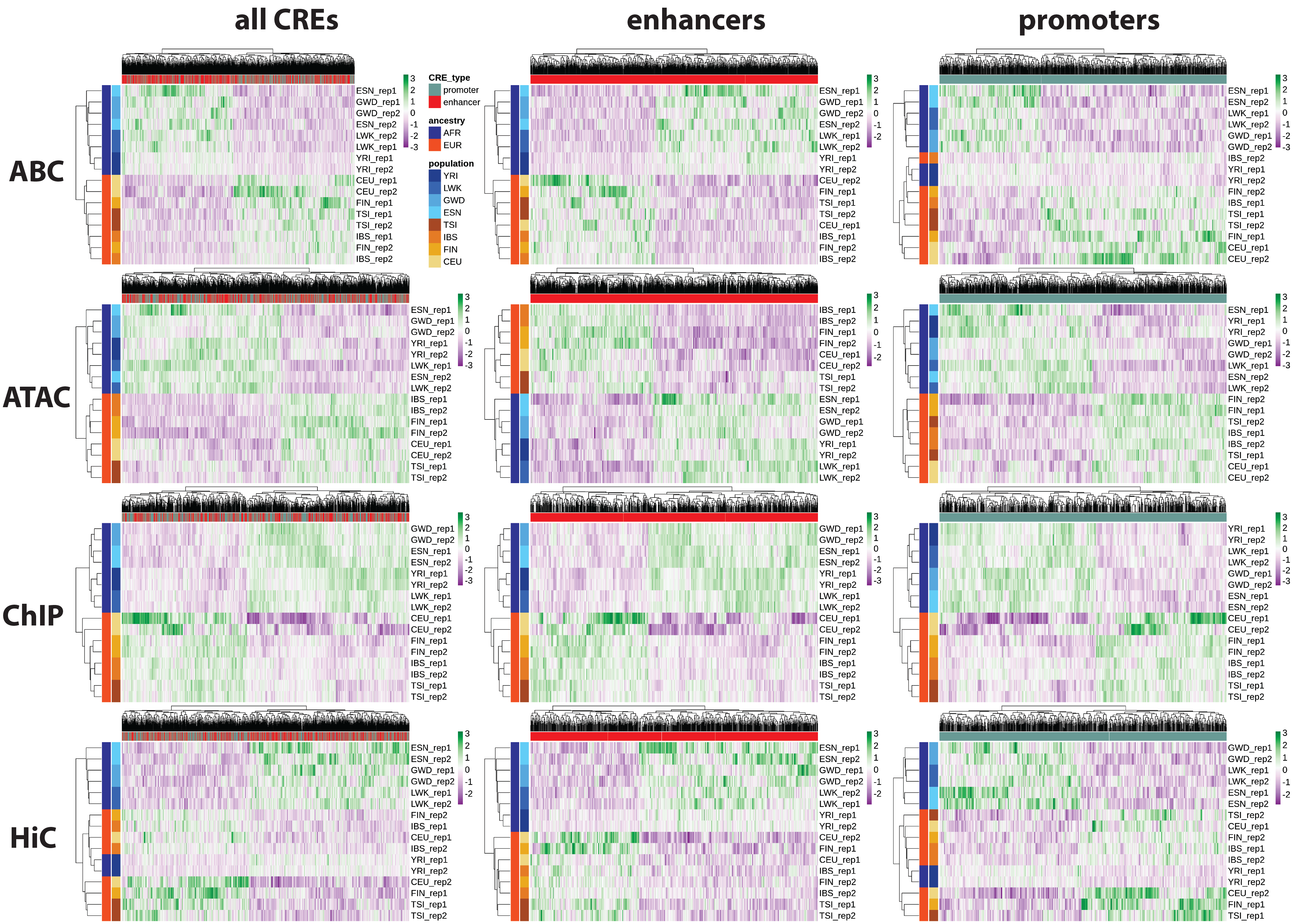
**

**Figure S8. Heatmaps of top diff-E-G pair scores (CEU-inclusive) with hierarchical clustering.** E-G pairs were subset first by diff-score P < 0.05, then by lowest P-value per gene, then by CRE type indicated by column names before performing PCA on each score type (row names).

**
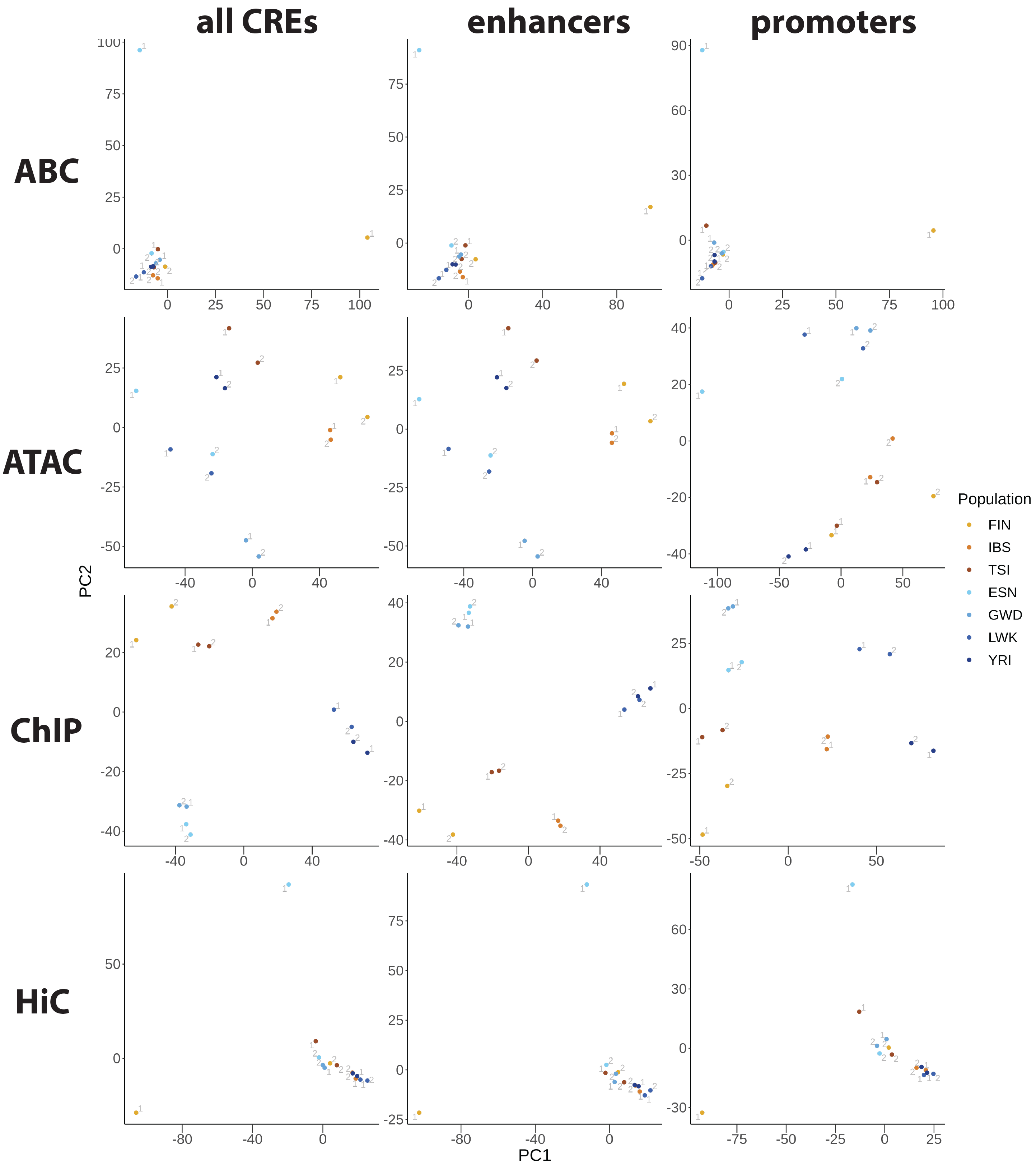
**

**Figure S9. PCAs of the 5,000 highest coefficient of variation E-G pair scores of each CRE type.** Replicate numbers are displayed next to each point.


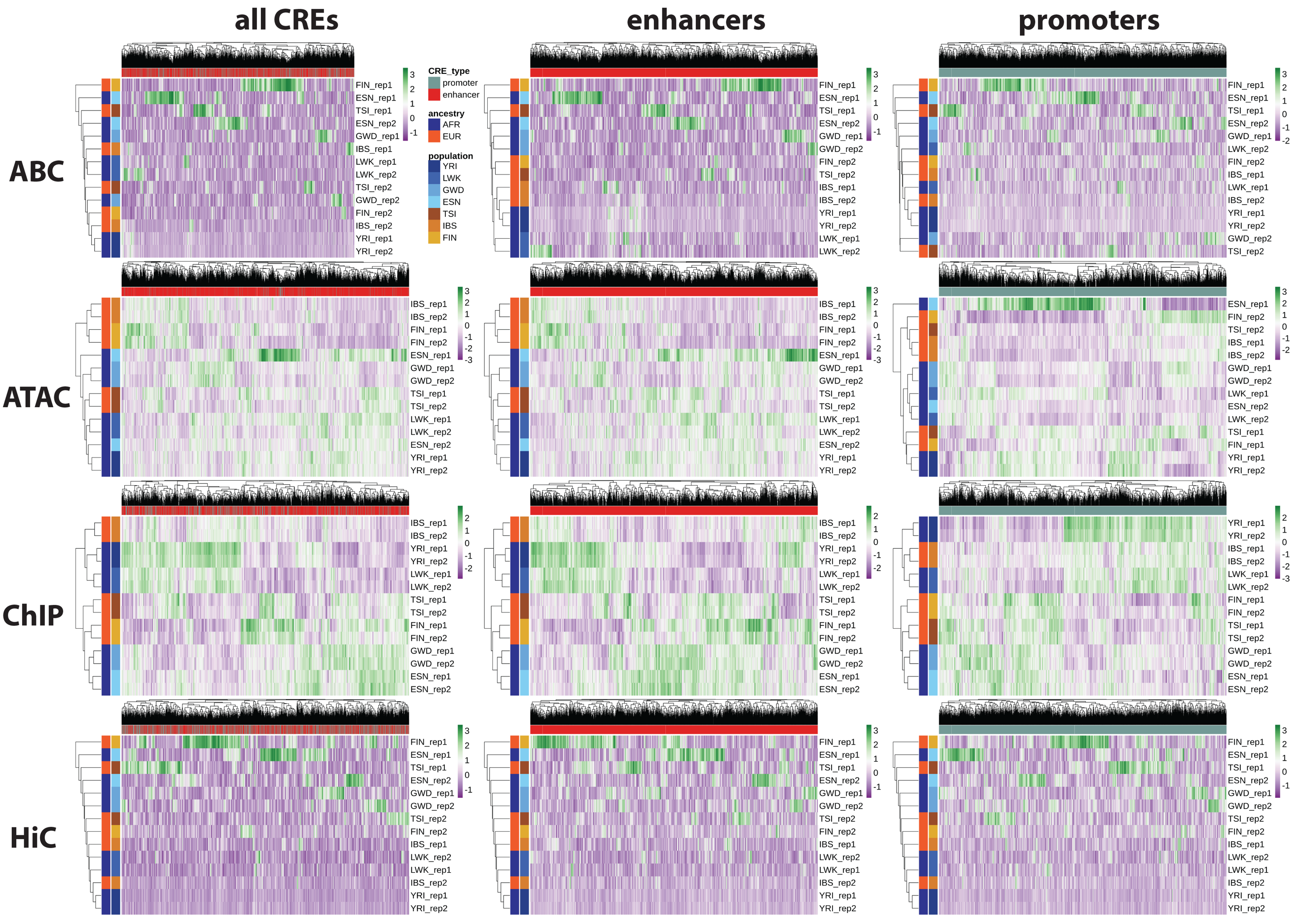


**Figure S10. Heatmaps of the 5,000 highest coefficient of variation E-G pair scores of each CRE type with hierarchical clustering.**

**
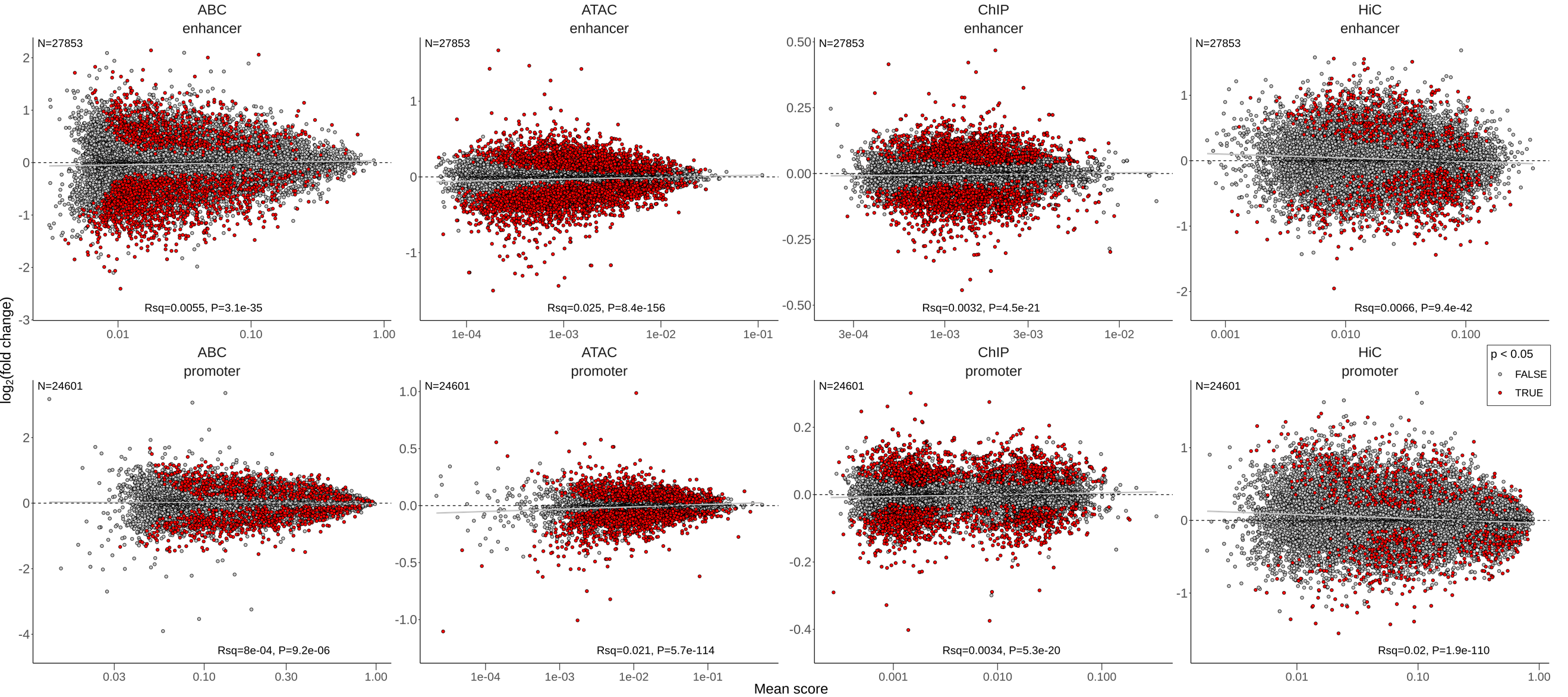
**

**Figure S11. MA-style plots of diff-CRE directionality.** The mean score (“A” scale) is plotted against the log_2_ ratio (“M” scale) or fold change of mean EUR score over mean AFR score for each CRE and score type. Red points are E-G pairs with diff-score P < 0.05.


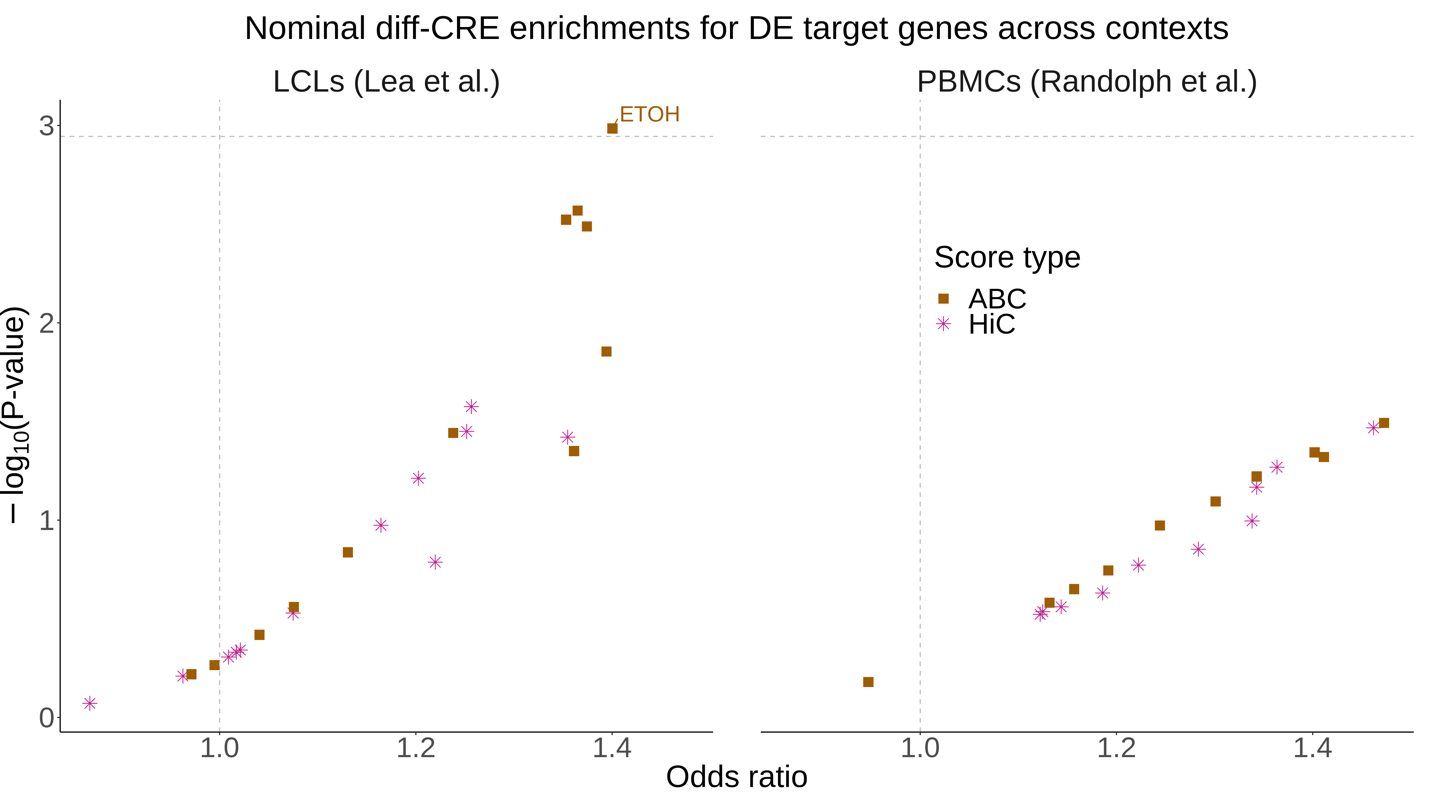


**Figure S12. Nominal diff-ABC and HiC enrichments for DE target genes across conditions and cell types.** See Fig. 2a,b legend. Horizontal dotted lines representing the Bonferroni corrected threshold are shown only for comparison and consistency with the main text figures, since these were not hypothesis tests due to the high diff-ABC and HiC FDRs (0.52 and 0.87, respectively, at diff P < 0.05).

**
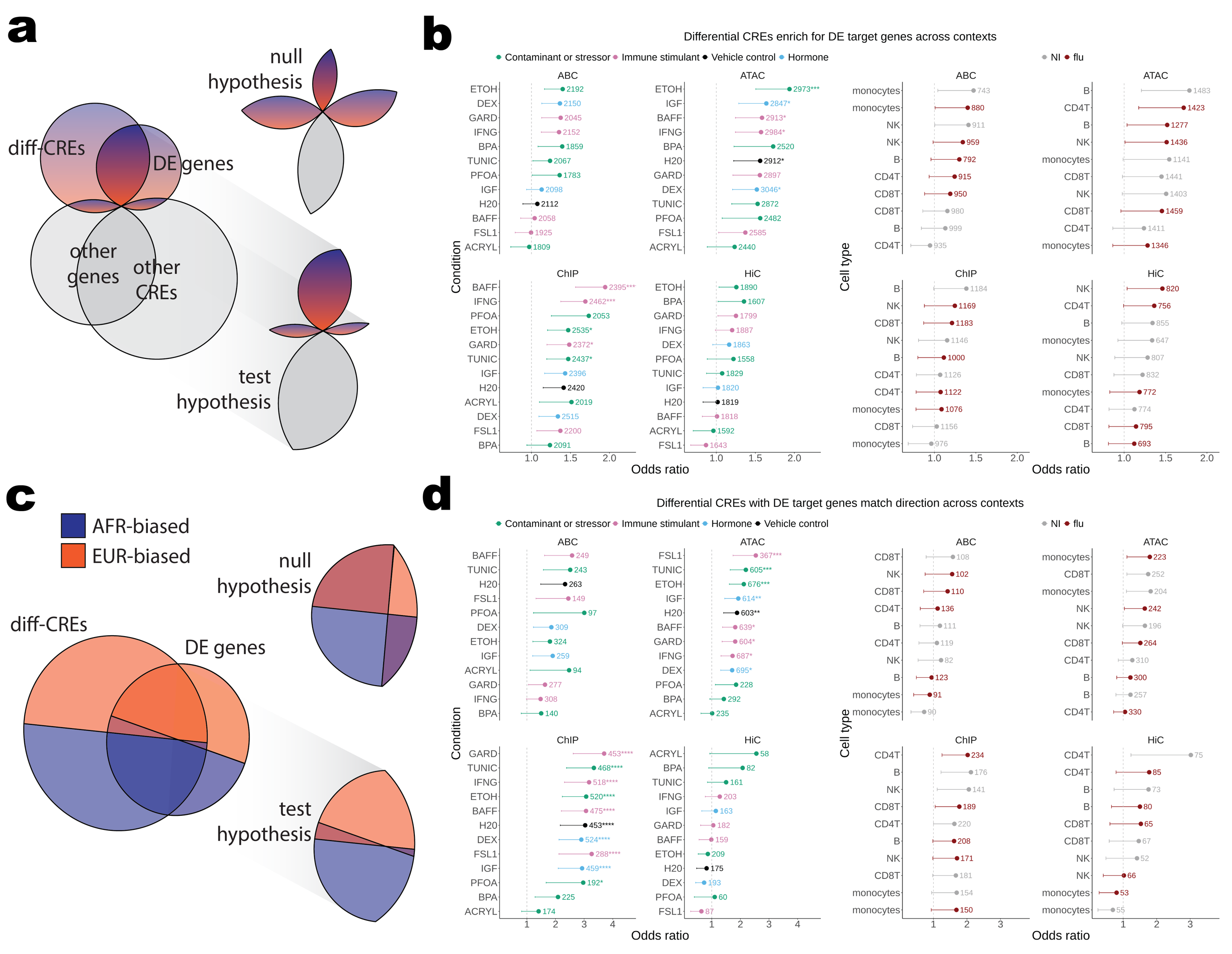
**

**Figure S13. Diff-CRE enrichments for DE across conditions and cell types.** See Fig. 2 legend, except here results of one-sided Fisher’s exact tests for DE gene enrichment in diff-CREs (b) and diff-CRE matching DE directionality (d) are plotted as odds ratios with error bars representing the lower bound of the 95% confidence interval. Since these are one-sided tests, the upper bound is infinity and is not shown. The total number of CREs used in each test is shown to the right of each odds ratio with asterisks indicating if the P-value passed multiple test correction (*, **, ***, and **** for Bonferroni-corrected P-value < 0.05, 0.005, 5x10^-4^, and 5x10^-5^, respectively). Nominal diff-ABC and HiC enrichments are shown for comparison only and are not included in Bonferroni correction, since these were not hypothesis tests due to the high diff-ABC and HiC FDRs (0.52 and 0.87, respectively, at diff P < 0.05).


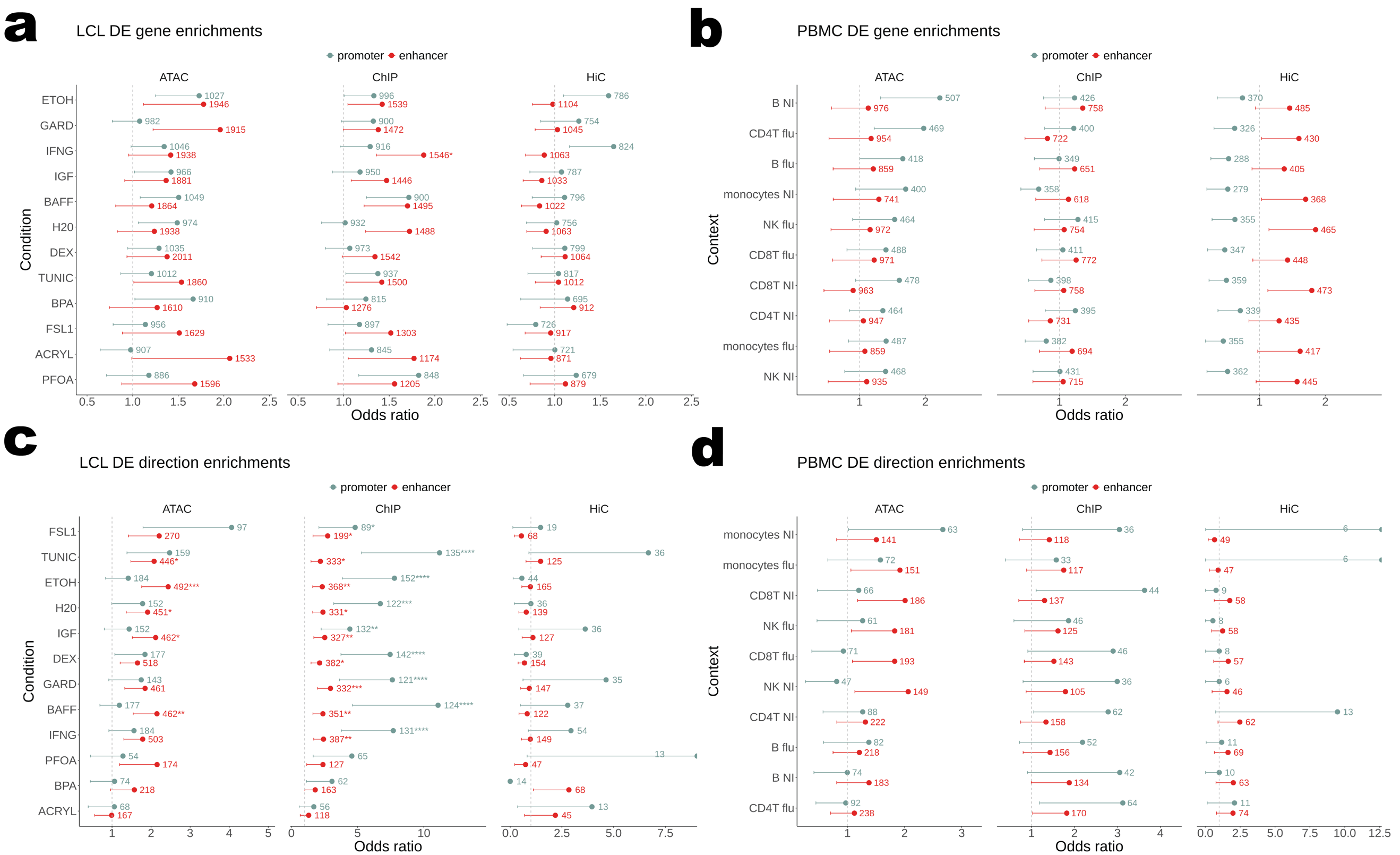


**Figure S14. Diff-CRE enrichments for DE across conditions and cell types by CRE type.** The same data used for tests in Fig. 2 and S12 were split into genes whose top diff-CRE was a promoter or enhancer and the same tests were performed on each, thus doubling the number of tests. See Fig. S12 legend. Nominal HiC enrichments are shown for comparison only and are not included in Bonferroni correction, since these were not hypothesis tests due to the high HiC FDR (0.87 at diff P < 0.05).


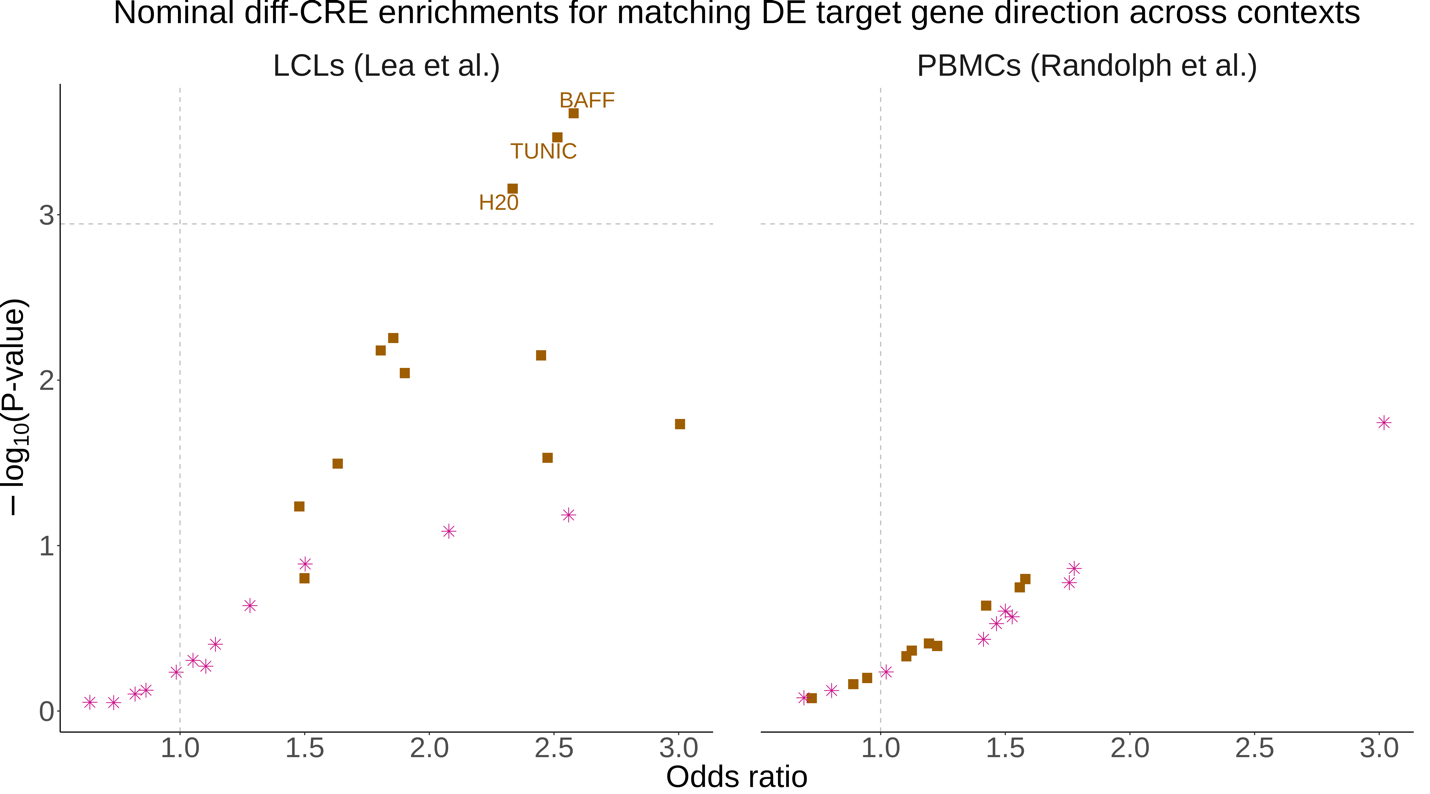


**Figure S15. Nominal diff-ABC and HiC enrichments for matching DE target gene direction across conditions and cell types.** See Fig. 2c,d legend. Horizontal dotted lines representing the Bonferroni corrected threshold are shown only for comparison and consistency with the main text figures, since these were not hypothesis tests due to the high diff-ABC and HiC FDRs (0.52 and 0.87, respectively, at diff P < 0.05).

**
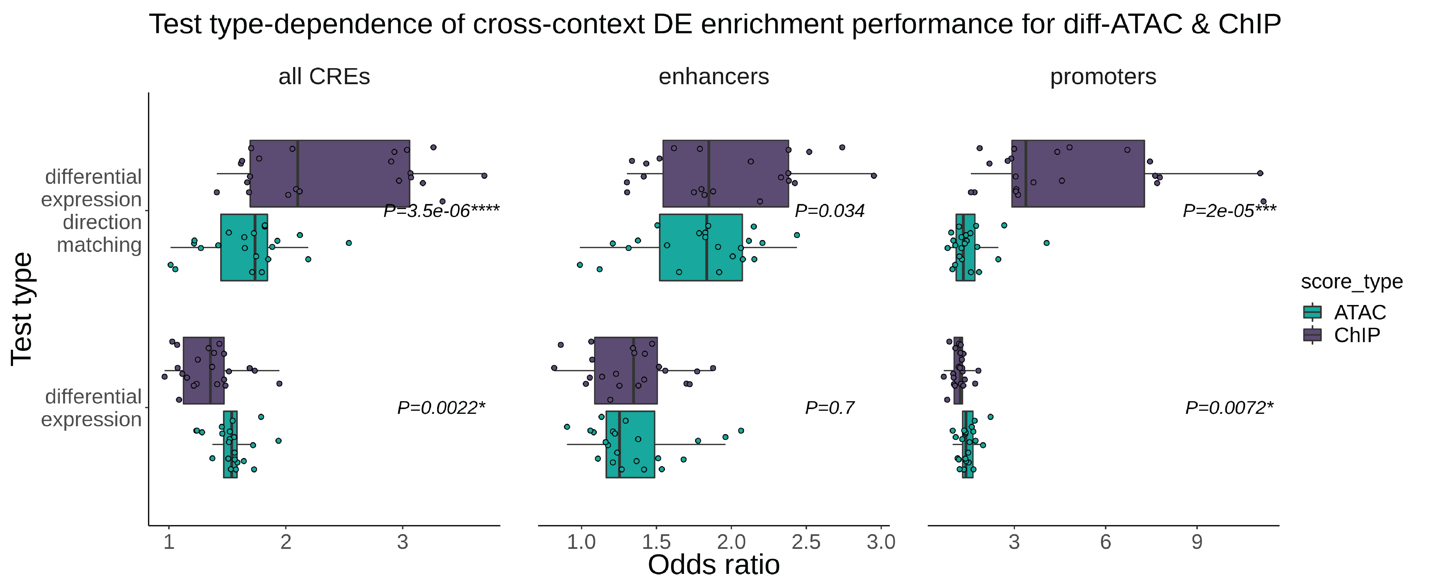
**

**Figure S16. Comparison of odds ratios between diff-ATAC and diff-ChIP from DE gene and matching DE directionality enrichment tests.** Odds ratios from tests in Fig. 2d are plotted as boxplots for each test type and CRE type. P-values from Wilcoxon tests on diff-ATAC versus diff-ChIP odds ratios in each category are shown. Asterisks indicate if the P-value passed multiple test correction (*, **, ***, and **** for Bonferroni-corrected P-value < 0.05, 0.005, 5x10^-4^, and 5x10^-5^, respectively).

**
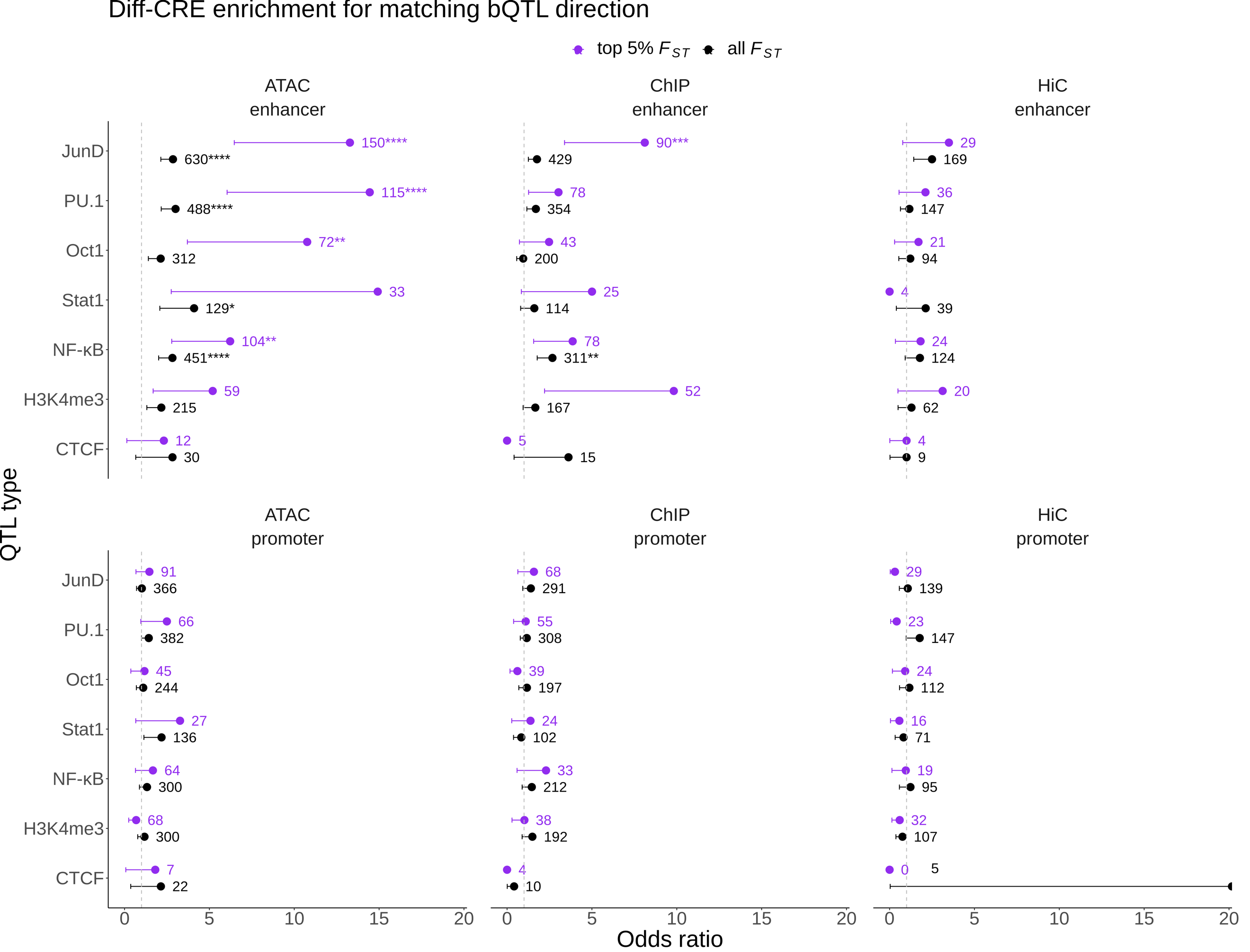
**

**Figure S17. Enrichments for direction matching between bQTL and diff-CREs are driven by enhancers.** See Fig. 3e legend.


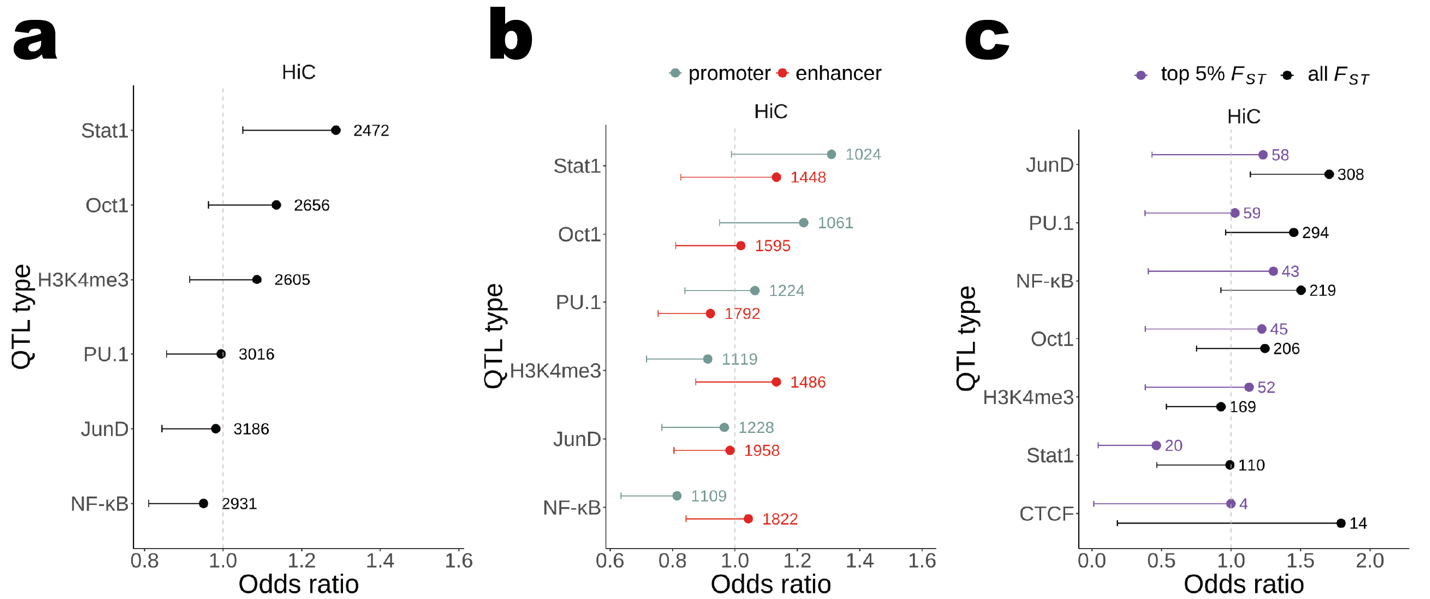


**Figure S18. Nominal diff-HiC bQTL enrichment test results. a)** See Fig. 3b legend. **b)** See Fig. 3c legend. **c)** See Fig. 3e legend.


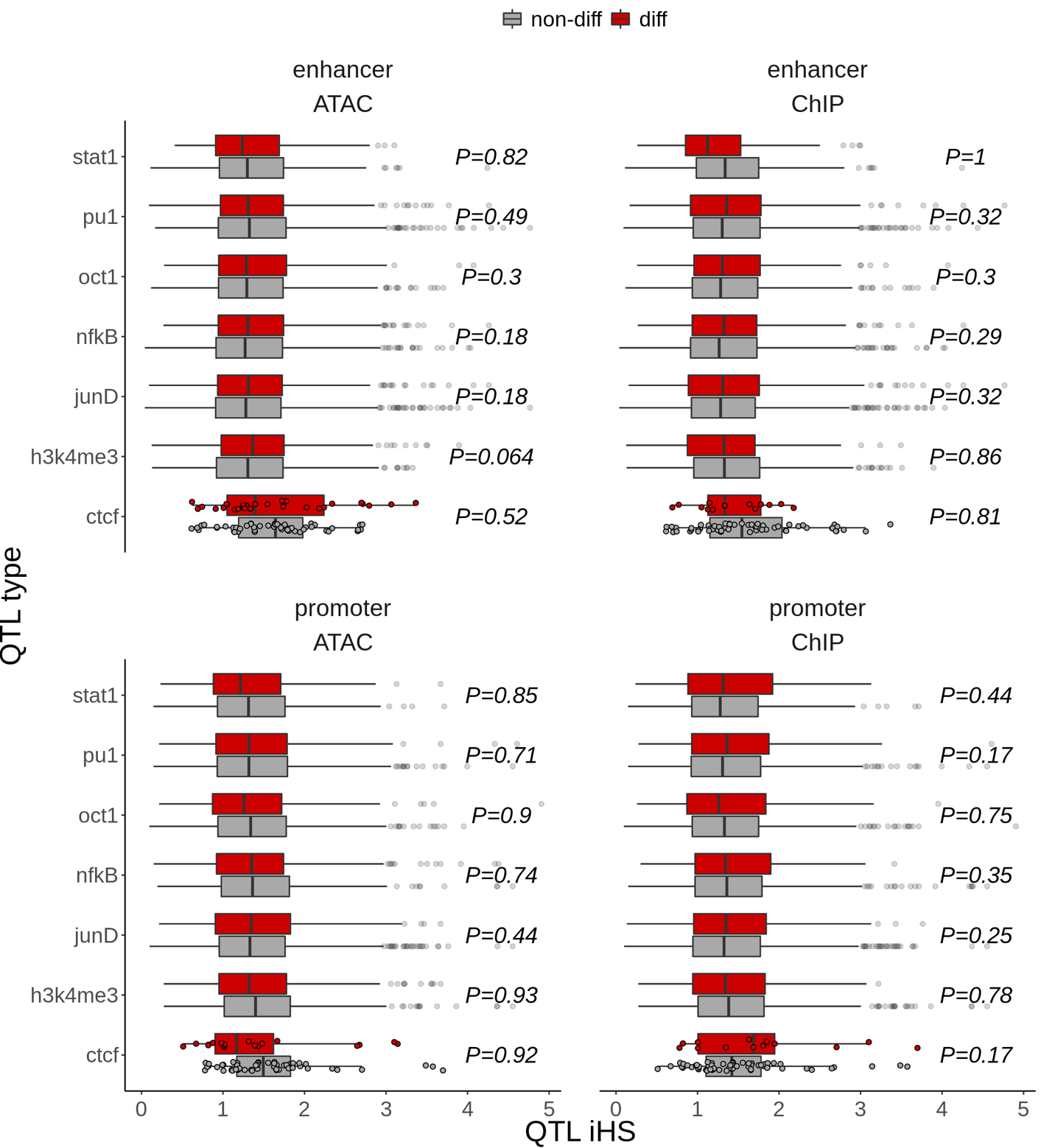


**Figure S19. iHS in diff- vs non-diff CREs. a)** iHS values of TF bQTL and H3K4me3 QTL are shown as boxplots for diff- and non-diff CREs of each displayed score type separated by if the QTL is in an enhancer (top) or promoter (bottom) analogous to Fig. 4a.

**
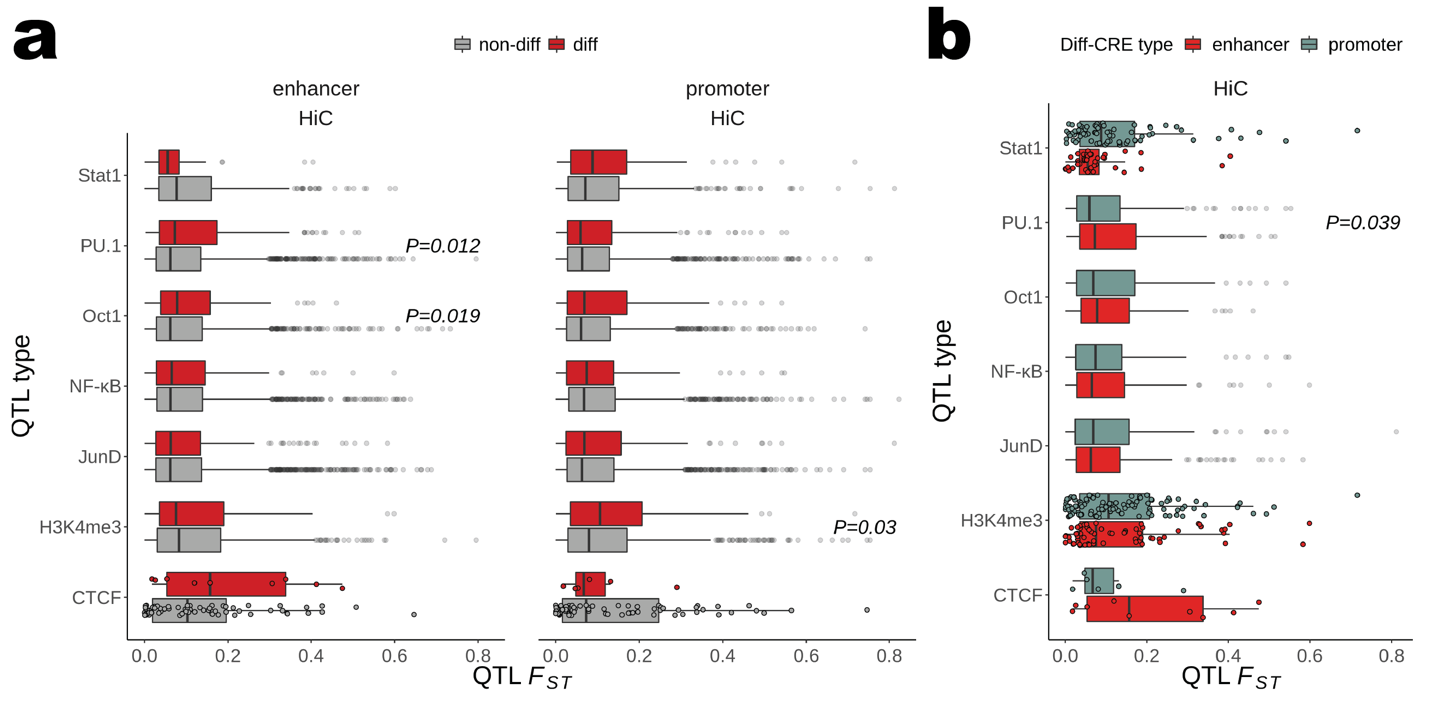
**

**Figure S20. Nominal diff-HiC bQTL ancestry divergence in enhancers versus promoters. a)** See Fig. 4a legend. **b)** See Fig. 4b legend.


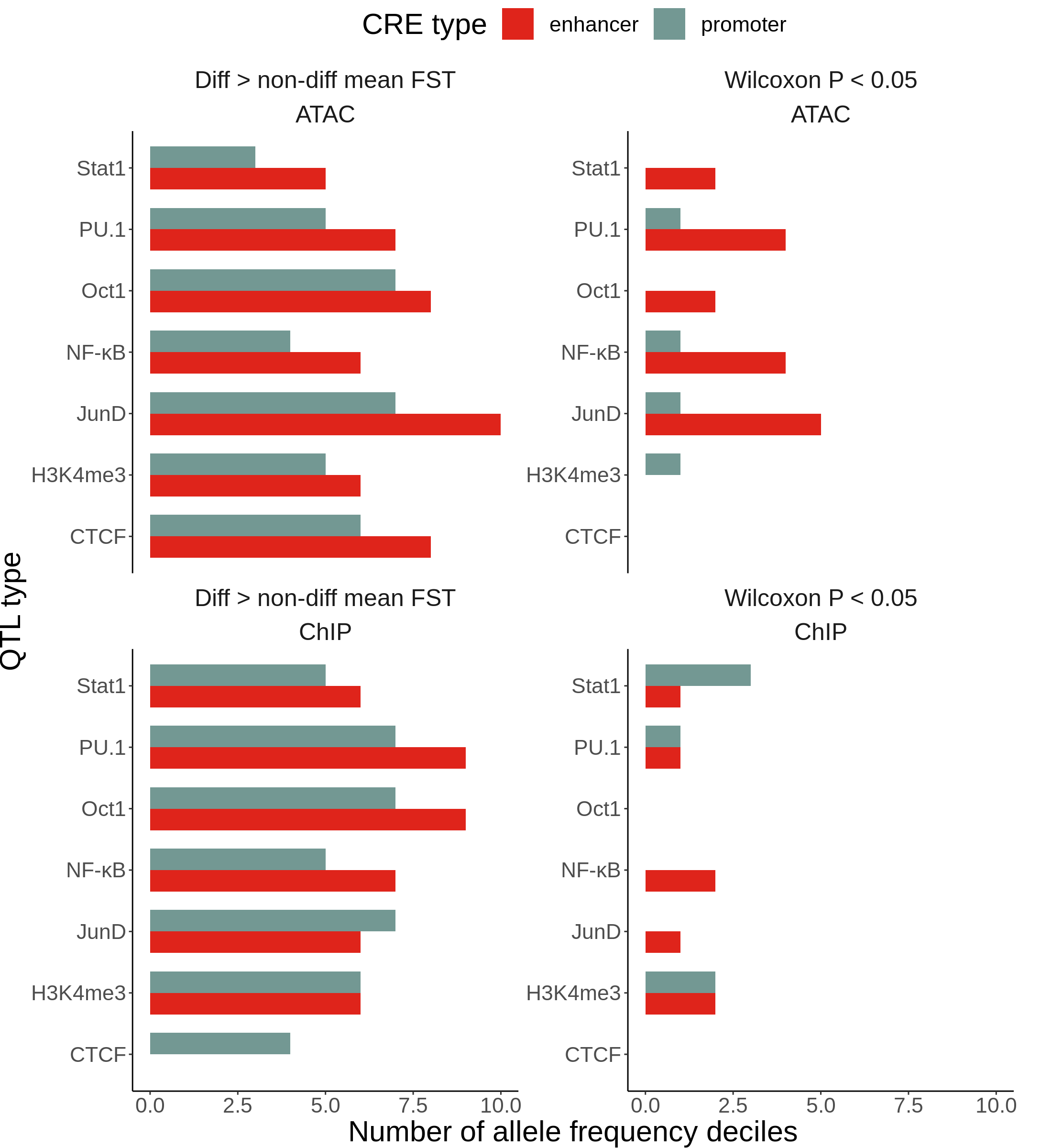


**Figure S21. Diff-activity bQTL ancestry divergence in enhancers versus promoters binned by allele frequency.** The number of allele frequency decile bins with greater mean *F_ST_* values of TF bQTL and H3K4me3 QTL in differential versus non-differential CREs is plotted grouped by CRE type (left). The number of allele frequency decile bins with nominally significant differences in *F_ST_* values of TF bQTL and H3K4me3 QTL between differential versus non-differential CREs by Wilcoxon tests within each bin is plotted grouped by CRE type (right).


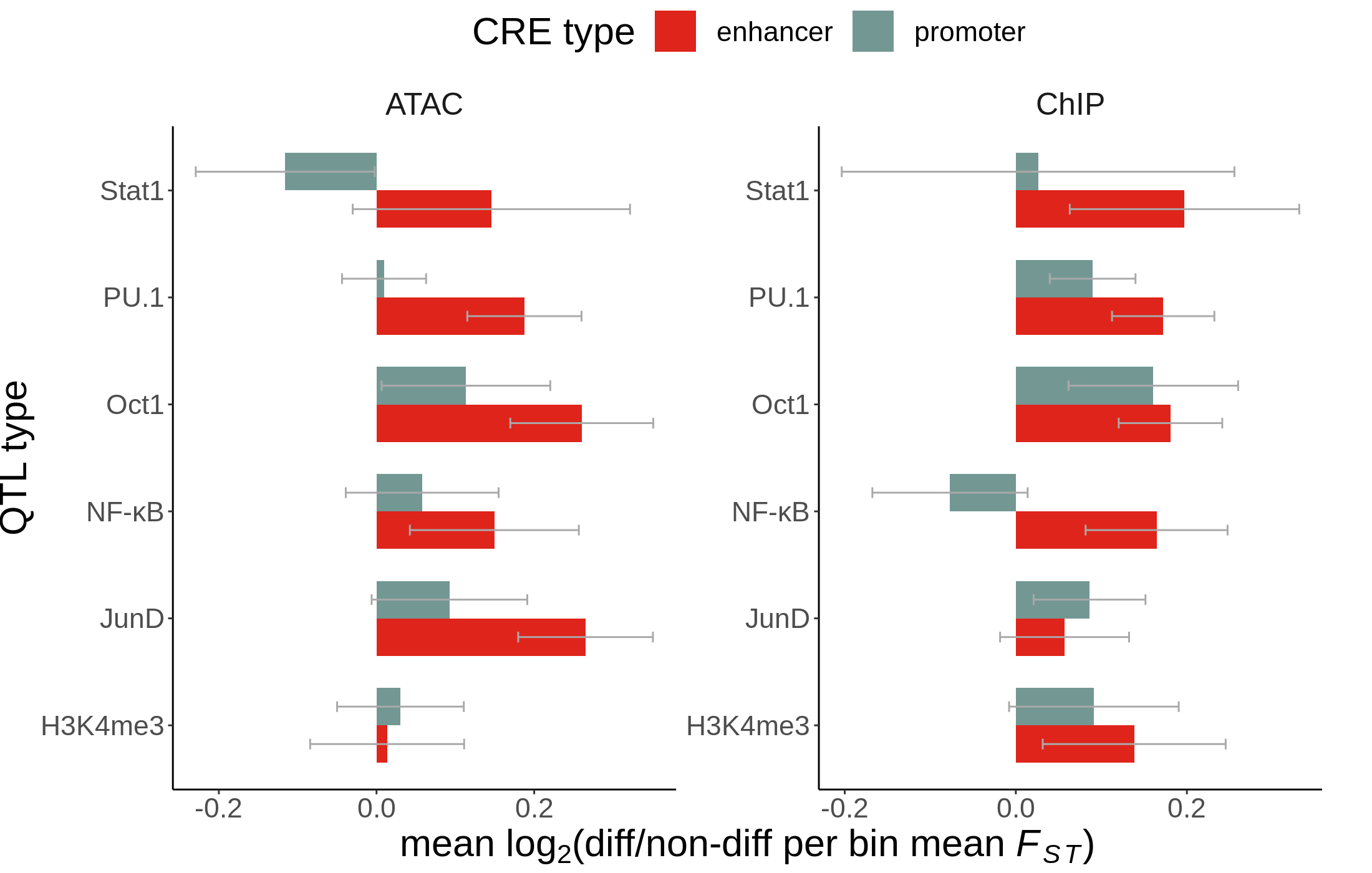


**Figure S22. Magnitude of diff-activity bQTL ancestry divergence in enhancers versus promoters binned by allele frequency.** The mean log_2_ fold-change in mean *F_ST_* values of TF bQTL and H3K4me3 QTL in differential over non-differential CREs is plotted with error bars representing the standard error of these means across allele frequency decile bins.

**
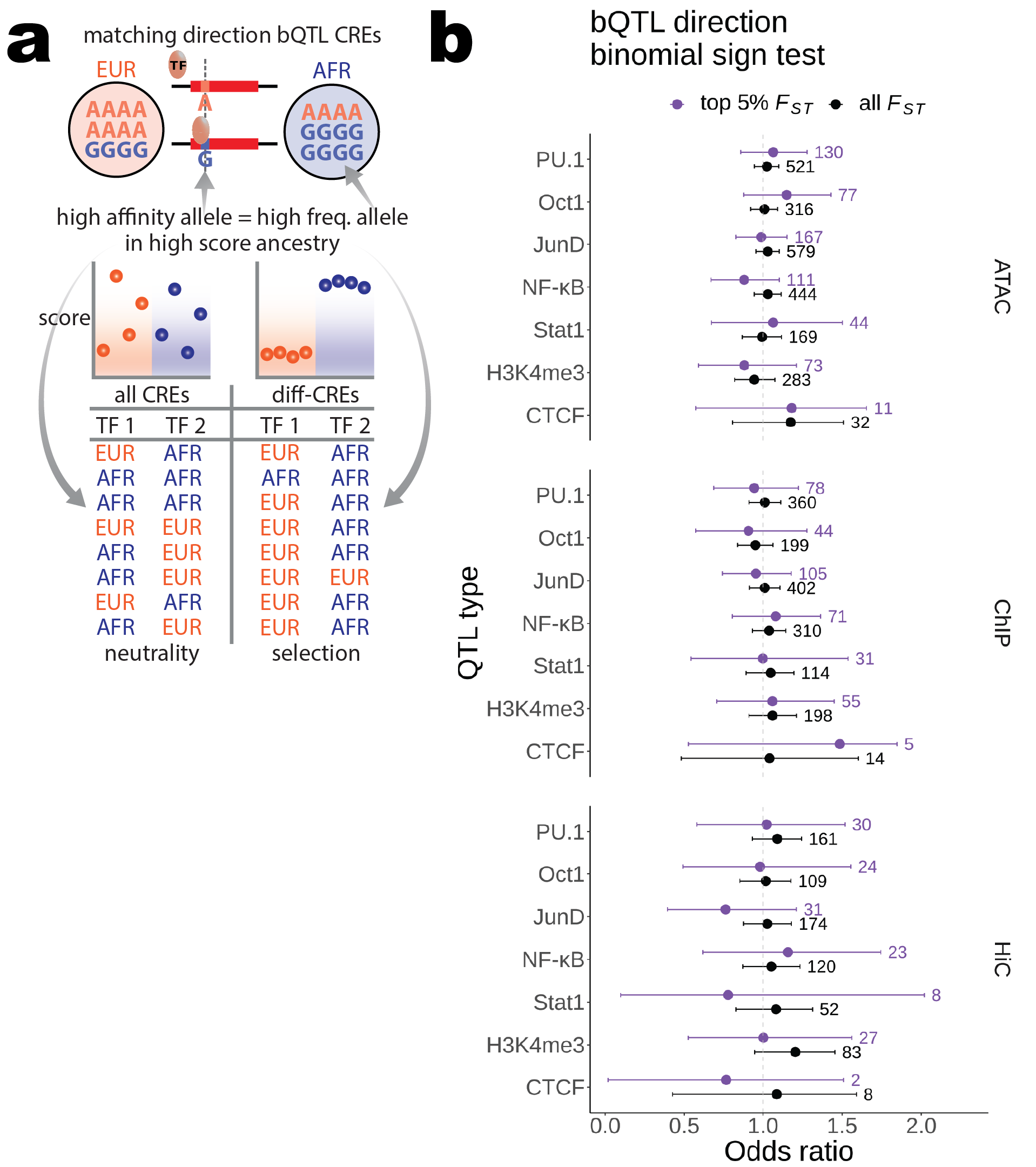
**

**Figure S23. Binomial sign test results on matching direction diff-CRE bQTL. a)** Schematic illustrating how the binomial sign test (results in (b)) was conducted. The hypothetical G allele at the top is the high affinity bQTL allele and is at higher frequency in AFR, which is also the ancestry with the higher ATAC, ChIP, or HiC scores for the CRE containing this bQTL. This matching direction bQTL CRE contributes one to the “AFR” count for “TF 2” in diff-CREs, whose binding sites therein are under directional selection for greater binding in AFR and/or reduced binding in EUR relative to the proportion of AFR matching direction bQTL CREs genome-wide (expectation under neutrality). **b)** Results of the two-sided binomial test described in (a) are plotted for each QTL type as odds ratios with error bars representing 95% confidence intervals. The total number of diff-CRE bQTL used in each test is shown to the right of each upper bound. None of the P-values pass multiple test correction, so no asterisks are displayed. Binomial test results were normalized to the background probability of success for visualization such that an odds ratio of one represents the background probability of success. Values greater than one indicate the TF has a greater proportion than expected of matching direction diff-CRE bQTL favoring greater binding in AFR.


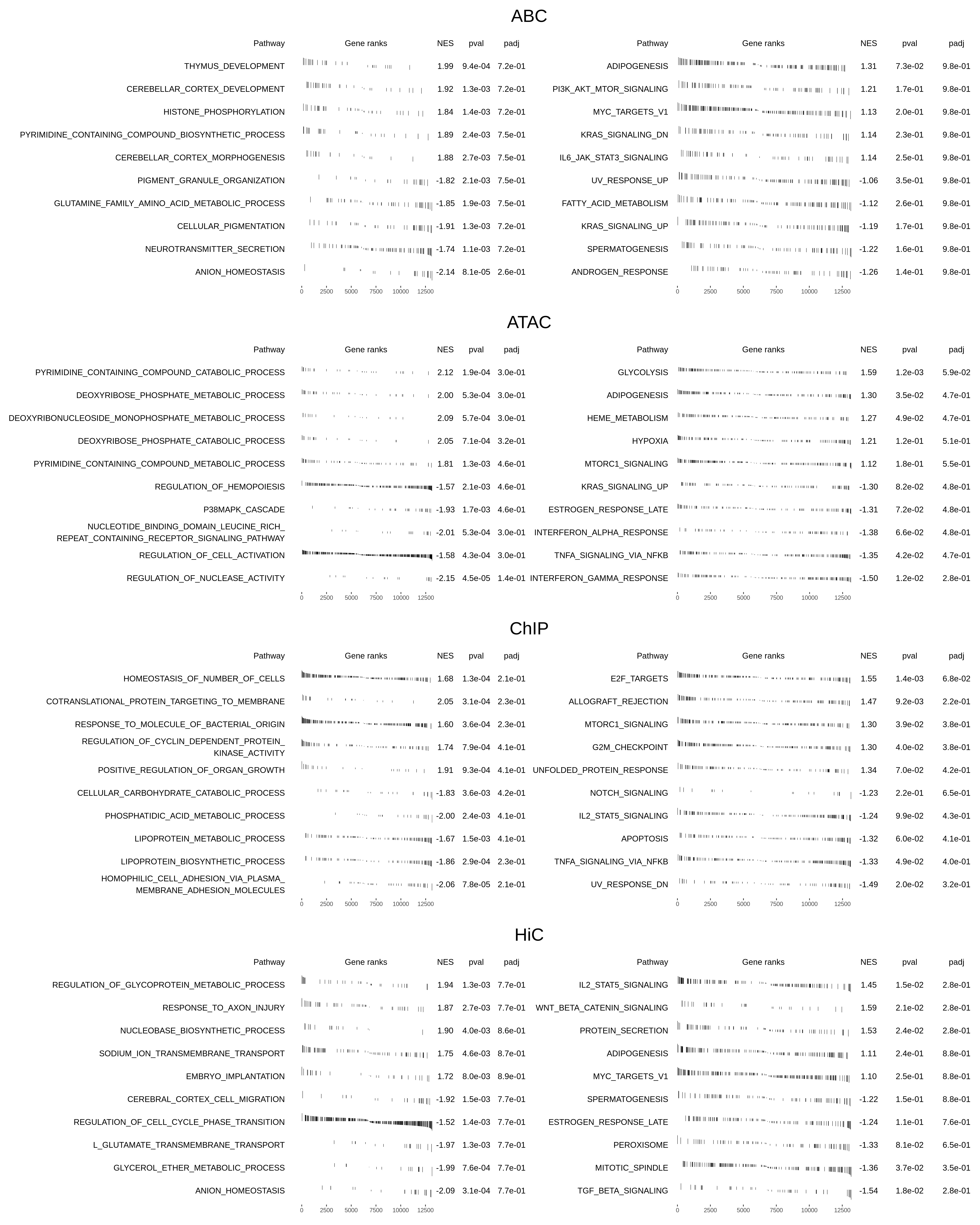


**Figure S24. Diff-CRE target gene directional pathway enrichment test results.** Results of gene set enrichment analyses from GO biological processes (left) and MSigDB Hallmark gene sets (right) are shown for each score type. A ranking statistic (see Methods), was used to rank all target genes by their most differential CRE from high EUR score to high AFR score. The top 5 processes or gene sets most enriched at the EUR (positive NES) and AFR (negative NES) ends of each list are displayed. Vertical black bars represent the value of the ranking statistic and location in the ranked list where a gene is in a given gene set. Abbreviations: pval = enrichment P-value, NES = normalized enrichment score, padj = Benjamini-Hochberg-adjusted P-value.

**Supplemental references**

1. Yuan Y, Tian L, Lu D, Xu S. Analysis of Genome-Wide RNA-Sequencing Data Suggests Age of the CEPH/Utah (CEU) Lymphoblastoid Cell Lines Systematically Biases Gene Expression Profiles. Sci Reports 2015 51. 2015 Jan 22;5(1):1–5.

2. Johnson KE, Voight BF. Patterns of shared signatures of recent positive selection across human populations. Nat Ecol Evol 2018 24. 2018 Feb 19;2(4):713–20.
