# Supplementary material for: Chromatin activity identifies differential gene regulation across human ancestries
Source: Genome Biol. 2024 Jan 15;25:21. doi: 10.1186/s13059-024-03165-2 (PMC10789071; doi:10.1186/s13059-024-03165-2)
Supplement: Supplementary file 7 — Additional file 7. Peer review history. [file 13059_2024_3165_MOESM7_ESM.docx]

**Review history**

**First round of review**

**Reviewer 1**

The authors study differences in epigenomic footprints of cis-regulatory activity between populations of European and African ancestries. They rely on previously published ATAC-seq data and newly generated HiChIP data to find differential cis-regulatory elements (diff-CREs) and test if the discovered ancestry-associated (diff-)CREs are statistically associated with differential expression of relevant genes between ancestries. The main take-away from the study is that there is a greater-than-random association between changes in expression and changes in chromatin accessibility. This is a useful contribution of the work, and to my knowledge this specific claim in the context of ancestries has not been made previously.

The work also presents many analyses that do not ultimately lead to any reliable findings (i.e., where null hypothesis could not be rejected), and while I am not against reporting such negative results, the authors may use their discretion to trim the narrative somewhat (perhaps through the use of supplementary notes), in the interest of better readability.

It seems that the HiChIP data was ultimately not particularly useful. The main claim(s) are equally or better made with existing ATAC-seq data. Also, the use of HiChIP data to get to ChIP-like data for H3K27ac comes off more as a "patch-job" of a failed test (i.e., the HiChIP data itself did not furnish the insights hoped for, so it was adapted into a surrogate of ChIP data, which did furnish some insights). Perhaps the paper could have been more to-the-point without the HiChIP data altogether.

The ABC scores used in their first methodology seem to have failed to provide any strong inferences. It is unclear why the authors chose to start with identification of "diff-ABC" CREs if they knew "ABC scores are designed to identify enhancer-gene pairs, but not the relative expression". This to me is one of the examples of a failed methodological choice and the authors may wish to remove that part of the presentation for better readability, unless they believe it serves a strong purpose in the context of their message.

I did not understand the rationale for redefining the ABC score by replacing the "HiC" contact component with the quantitative "HiChIP" signal. In my understanding, it makes sense to multiple "activity" by "contact frequency" as in the original ABC, but "HiChIP" is itself a function of both contact frequency and activity (since H3K27ac ChIP is related to activity). This likely distorts the originally intended semantics of the ABC score.

The authors should clarify why the formulas for "ChIP score" and "HiC score" are the correct way to "decompose" the HiChIP scores/data.

Examination of differential CRE activity vis-à-vis ancestry-divergent variants leads to an interesting statistical finding involving enhancers but not promoters (Fig 3c). Do the diff-CREs have greater "coverage" in the enhancer class compared to the promoter class? Could this be part of the reason why promoters do not show significant enrichments?

I believe the conclusion (line 294) that "many ancestry differences in CRE activity could be associated with differences in binding of specific TFs in cis, rather than differences in TF expression levels in trans" could be misinterpreted by the reader. The "many" here simply refers to more than random expectation, which is low. Similarly, the wording "Having established ancestry-dependent cis differences in TF binding as a likely mechanism for ancestry-associated differential CRE activity, …" can be misinterpreted by a reader. The analysis has found some evidence that cis-differences in TF binding may be one of the mechanisms for ancestry-associated differential CRE activity, not that it is a "likely" mechanism. Also, the "rather than differences in TF expression levels in trans" is not something that has been tested here.

The purport of the paragraph from line 321 to 340 is unclear. For instance, while the finding that "JunD, NF-κB, PU.1, and Oct1 bQTL have higher FST in diff-ATAC than in non-diff-ATAC enhancers" is syntactically clear, it is hard for the reader to judge what to make of it. Some of the other tests do not cross significance levels so no conclusion can be drawn.

Regarding claims about promoter, e.g., "enhancers rather than promoters display the strongest signs of selection associated with sites of differential transcription factor binding.", could the insignificant p-values in tests with promoters arise because differential promoter activity is harder to detect (less correlated) with differential ATAC-seq? If the detection methodology (diff-ATAC) is biased against detecting promoters for some reason, then the claims made here regarding differences in enhancer/promoter biology are not conclusive.

Minor comment:
In the definition of "ChIP-score_E,G", is it true that the \sqrt{H_G} term cancels out in the numerator and denominator, leaving the formula as one proportional to \sqrt{H_E}? What is the rationale for this relationship?

**Reviewer 2**

In this manuscript, Pettie et al. examined the potential impact of ancestry-specific selection acting on chromatin activity, TF binding, and chromosomal looping. The authors build a strong case regarding the evolutionary and functional importance of regulatory DNA, and topics covered in their manuscript lie within the scope of Genome Biology. Although the text is a little dense in parts, the quality of the writing in this manuscript is acceptable. Similarly, the figures are serviceable. I liked the idea of looking at the level of chromatin to identify potential targets of adaptation that may have been missed by traditional DNA-based scans. Interestingly, the authors observe different signatures with respect to ancestry for ATAC-seq, ChiP, and HiC data. The innovation of this study and it's potential impact make it a good fit for a journal with an impact factor like that of Genome Biology. However there are three key technical and methodological issues that dampen my enthusiasm for this manuscript.

First, all of the experiments used lymphoblastic cell lines (LCLs). How robust are the primary findings of this paper to the use of LCLs? Do ancestry differences in ATAC, ChIP, and HiC scores occur in other types of cells? I understand the difficulties re: obtaining other types of cells from ancestrally diverse populations. However, the authors need to build a stronger case that their results are robust to the choice of cell lines - especially since they are making the provocative claim that differential gene regulation across ancestries is modulated by chromatin activity. For example, how similar are ATAC,ChIP, and HiC scores for LCLs and whole blood of individuals who have the same ancestry? Note that buttressing this point need not require a complete set of additional experiments. Expansion of the text and inclusion of multiple supportive citations might end up being sufficient.

Second, data were quite noisy across replicate pairs of experiments. Given that no less than 4 out of 16 key datapoints (2xCEU and 1xESN and 1xFIN) appear to be outliers, additional replicates are required. If strong claims about differences across ancestries are to be made it is imperative that that the results within each ancestry show greater consistency.

Third, the tests of population differentiation and natural selection can be enhanced. For example, Fst statistics are know to be lower for rare alleles that are due to recent mutations (think of a comparison between a population that has an allele frequency of 0 vs. an allele frequency of 0.001). Are the sets of SNPs in Figure 4A (non-diff vs. diff) and Figure 4B (promoter vs. enhancer) frequency-matched? If not, some sort of frequency binning (by allele age or global allele frequencies in the 1KGP) might be advisable. To strengthen the claims that natural selection contributes to differentiation between ancestries it would be good to examine other types of selection statistics, e.g., iHS or any of the statistics included in Abrahim et al. 2023 (https://doi.org/10.1371/journal.pgen.1010494).

Other technical details of this study seem to be in well-thought-out. The choice of a pooled study design to examine four African (ESN, GWD, LWK, and LWK) and four European (CEU, FIN, IBS, and TSI) ancestry populations is acceptable. Similarly, the additional methodological choice to resize ATAC-seq peaks to 500bp and retaining the top 150k peaks by read count seems reasonable. The methods used to reduce allelic mapping bias of H3K27ac HiChIP.

An additional consideration involves the recently published document by the National Academies of Science, Engineering and Medicine (Using Population Descriptors in Genetics and Genomics Research - A New Framework for an Evolving Field). While I personally disagree with Conclusion 5-4 in the NASEM document: "Given that underlying cellular and physiological mechanisms are expected to be universal among humans, the default practice in such studies should be to not use any descent-associated population descriptors," Pettie et al. should be cognizant that their manuscript is likely to receive intense critical attention. That said, as illustrated by Lea et al. 2022, Randolph et al. 2021, and Pettie et al. (GBIO-D-22-01655)… differential gene expression and chromatin can indeed differ across ancestries.


Additional comments:

I appreciated the honesty of the authors regarding the limitation of some of their marginally significant results (e.g., the impact of promoter or enhancer activity on ancestry differential expression).

Page 6, line 118: This is an interesting idea, but wouldn't selecting acting on the level of chromatin activity also leave some sort of signature in terms of DNA? The authors are encouraged to expand upon this section of the introduction. What I'm thinking about here are situations where putative signatures of selection on the level of chromatin activity and the level of DNA sequences are incongruent. How to interpret such signals? Note that the expansion of text related to this topic may be better suited for a discussion point.

Page 9, line 192: I'm not a fan of removing both CEU replicates, as these constitute two out of 16 total data points.

Some figure legends are overly long (e.g., Figure 1 and Figure 3). The authors are encouraged to put some of these details in the main text or to split figures into a smaller number of panels.

Page 18 (Figure 1): Something is strange in Figure 1C. Why do the ESN replicate pairs and the FIN replicate pairs not cluster together in the ABC and HiC sub-panels?

Page 20 (Figure 2). I'm not a big fan of the schematics shown in Figure 2 (the oddly shaped quadrants are a bit non-intuitive).
The main text does a much better describing the approach the authors took. That said, graphical abstracts of methods can be useful. The authors are encouraged to find alternative ways of representing the approaches described in panel C of this figure.

Page 23 (Figure 4). P-values for all comparisons should be shown

Page 31, line 783: It might be good to provide additional justification (a citation perhaps) regarding the denominator in the GO Analysis equation that was use to rank genes. Even though it seems appropriate, it would be good to state why this particular equation was used.

**Authors’ response to reviewers**

Once again, this well written manuscript by Pettie et al. examines whether there is evidence of ancestry-specific natural selection on chromatin activity, transcription factor binding, and chromosomal activity. Despite the limitations of their dataset, the authors have done an admirable job of blending population genetic analyses with chromatin activity data from lymphoblastoid cells. Overall, the authors have done a reasonable job of responding to reviewer comments (including my own). Changes made to the text have resulted in an improved, more comprehensive manuscript. However, there is one key fix that needs to be made to warrant publication in Genome Biology (dealing with outlier samples).

Although I am generally satisfied with this revised manuscript, there is still a small chance that the continental ancestry-level differences that were observed could due to some other factor (as only a small number of samples are described in Figure 1C. In their response to reviewer comments the authors bring up the good point that both outlier CEU replicates were older samples (dating back to the 1970s). What are the ages of all of the other samples? Is there any known reason why the FIN rep1 and ESN rep1 samples were outliers for ABC and HiC? For the sake of completeness the authors are highly encouraged to included a supplemental table listing additional features/metadata related to all 16 of the samples. The idea here is to rule out batch effects or other possible causes of differences in chromatin activity between AFR and EUR individuals (like differences in sample preparation or ages of LCL cells).


Additional notes:

I am satisfied with the author's response to my point about the limitations of LCLs, including the revisions made to the discussion section.

The authors have done a good job of enhancing their tests of natural selection. I am glad that additional figures (S20-S21) and text now highlight that their results are not driven by allele frequencies between tested CRE types. Although the iHS tests ended up having negative results (lack of major differences in iHS between non-diff and diff-CRES), for completeness it would be good to include the QTL type x QTL iHS plot as an additional supplemental figure.

Lines 260-264: Please add additional statistical details in these sentences. What were the p-values and tests that were applied? How much did DE genes over lap more than expected by chance?

# Reviewer #1:

The authors study differences in epigenomic footprints of cis-regulatory activity between populations of European and African ancestries. They rely on previously published ATAC-seq data and newly generated HiChIP data to find differential cis-regulatory elements (diff-CREs) and test if the discovered ancestry-associated (diff-)CREs are statistically associated with differential expression of relevant genes between ancestries. The main take-away from the study is that there is a greater-than-random association between changes in expression and changes in chromatin accessibility. This is a useful contribution of the work, and to my knowledge this specific claim in the context of ancestries has not been made previously.

The work also presents many analyses that do not ultimately lead to any reliable findings (i.e., where null hypothesis could not be rejected), and while I am not against reporting such negative results, the authors may use their discretion to trim the narrative somewhat (perhaps through the use of supplementary notes), in the interest of better readability.

We thank the reviewer for this suggestion and agree that the narrative would benefit from some trimming. Below we describe the specific adjustments we have made to the main text and supplementary notes accordingly.

It seems that the HiChIP data was ultimately not particularly useful. The main claim(s) are equally or better made with existing ATAC-seq data. Also, the use of HiChIP data to get to ChIP-like data for H3K27ac comes off more as a "patch-job" of a failed test (i.e., the HiChIP data itself did not furnish the insights hoped for, so it was adapted into a surrogate of ChIP data, which did furnish some insights). Perhaps the paper could have been more to-the-point without the HiChIP data altogether.

We thank the reviewer for pointing out the lack of clarity around the utility of the HiChIP data. These data were instrumental in defining our set of 50,478 candidate enhancer-target gene pairs according to the method described in Fulco et al. (2019) (see “*Jointly estimating Activity and Contact with HiChIP*” under the “Alternative methods to estimate Activity and Contact in the ABC model” section of Supplementary Methods). Their use of ABC scores computed from “quantitative HiChIP signal” and DNase hypersensitivity sequencing (DHS-seq) data achieves similar accuracy to those computed from a combination of DHS, H3K27ac ChIP-seq, and HiC data in predicting enhancer-target gene pairs in K562 (see their Extended Data Fig. 3d, also included for convenience in response to a later comment below). Our application of this method to define enhancer-gene pairs using ABC score thresholds (0.015 for enhancers and 0.1 for promoters) was a critical step prior to extracting the activity components from ABC scores to link regulatory elements differential activity to target gene differential expression. We have adjusted the main text to emphasize the utility of the HiChIP data in this regard (lines 237-240):

“Importantly, although we observe little-to-no differential HiC signal between ancestries, this component was critical in defining enhancer-gene pairs to test, as each pair must have at least one HiChIP read connecting the two elements to have a non-zero ABC score.”

The ABC scores used in their first methodology seem to have failed to provide any strong inferences. It is unclear why the authors chose to start with identification of "diff-ABC" CREs if they knew "ABC scores are designed to identify enhancer-gene pairs, but not the relative expression". This to me is one of the examples of a failed methodological choice and the authors may wish to remove that part of the presentation for better readability, unless they believe it serves a strong purpose in the context of their message.

We thank the reviewer for this recommendation to improve narrative. We initially aimed to test for evidence of selection on both activity levels and contact frequency simultaneously and believe that the lack of evidence for selection on contact frequency is noteworthy; however, we agree with the reviewer and have moved the “diff-ABC” analysis to the Supplementary Information in Additional File 1 and have streamlined the narrative in the first section of our results toward the new section title “Differential CRE activity is linked to differential expression between ancestries” (line 145).

I did not understand the rationale for redefining the ABC score by replacing the "HiC" contact component with the quantitative "HiChIP" signal. In my understanding, it makes sense to multiple "activity" by "contact frequency" as in the original ABC, but "HiChIP" is itself a function of both contact frequency and activity (since H3K27ac ChIP is related to activity). This likely distorts the originally intended semantics of the ABC score.

We appreciate the reviewer’s concern around the use of HiChIP data to calculate ABC scores. As indicated in our previous response addressing the utility of the HiChIP data, ABC scores were computed using HiChIP data in the original ABC paper and achieved similar performance to those reported in the main text of that study (see their Extended Data Fig. 3d, included below for convenience) [1]. We acknowledge this observation requires familiarity with the Extended Data, and have adjusted the main text to highlight it (lines 162-164):

“H3K27ac HiChIP was shown to perform at least as well as H3K27ac ChIP-seq and HiC assayed separately when used in ABC scores adapted for this data type [1].”

**Extended Data Fig. 3 | Investigating components of the ABC score. d,** Precision-recall curves for the ABC model using H3K27ac HiChIP. ABCDHS x H3K27ac Hi-ChIP corresponds to a predictive model whose score is proportional to the DHS signal at the candidate element multiplied by the H3K27ac Hi-ChIP signal between the element and gene promoter (see Supplementary Methods). ABCH3K27ac Hi-ChIP is the same as above but only uses the existence of the DHS peak as opposed to the quantitative signal in the DHS peak. H3K27ac HiChIP HiCCUPS Loops is the HiCCUPS loop calls derived from the H3K27ac HiChIP experiment (see Supplementary Methods). ABC corresponds to ABCsqrt(DHS x H3K27ac) x Hi-

C. These results suggest that the ABC score computed using H3K27ac HiChIP data is an effective predictor of regulatory enhancer-gene connections.

The authors should clarify why the formulas for "ChIP score" and "HiC score" are the correct way to "decompose" the HiChIP scores/data.

We thank the reviewer for highlighting this need for additional explanation and have expanded the “*Score computation”* section of the methods to include more detail behind our rationale for using the VC-sqrt to split HiChIP into signal from the ChIP enrichment versus contact enrichment steps of the protocol (lines 720-729):

“VC-sqrt normalization is commonly applied to HiC data for comparison of contact frequencies across samples since the assumption of equal visibility is reasonable when considering data generated from proximity-based ligation alone (*i.e.*, without ChIP). When applied to HiChIP, the VC-sqrt measures the difference in visibility between interacting regions relative to one another within a sample that is due to the levels of H3K27ac present at each region. Thus, when normalized by the sum of this signal across all elements within 5 Mb of the target gene, the resulting ChIP score reflects the contribution of H3K27ac levels to an ABC score. We can then use VC-sqrt normalization to estimate the contact frequency between each element independent of H3K27ac levels and extend this to compute the HiC component of an ABC score as follows…”

Examination of differential CRE activity vis-à-vis ancestry-divergent variants leads to an interesting statistical finding involving enhancers but not promoters (Fig 3c). Do the diff-CREs have greater "coverage" in the enhancer class compared to the promoter class? Could this be part of the reason why promoters do not show significant enrichments?

We appreciate the reviewer’s comment about the potential effects of coverage differences between promoters and enhancers on our findings. In fact, promoters have greater coverage than enhancers in both our ATAC and HiChIP data, so our finding that differential activity CRE enrichments for bQTL are driven by enhancers (Fig. 3b-c) is conservative when considering this coverage difference. We have adjusted the main text to highlight this and added Fig. S2 to Additional File 1 to illustrate these coverage differences (lines 300-301):

“This was despite greater coverage at promoters than at enhancers in both ATAC and HiChIP data (Fig. S2a-b, Additional file 1).”

Please see also our response to the comment below regarding the ability of both methodologies to detect differential promoter versus enhancer activity.

I believe the conclusion (line 294) that "many ancestry differences in CRE activity could be associated with differences in binding of specific TFs in cis, rather than differences in TF expression levels in trans" could be misinterpreted by the reader. The "many" here simply refers to more than random expectation, which is low. Similarly, the wording "Having established ancestry-dependent cis differences in TF binding as a likely mechanism for ancestry-associated differential CRE activity, …" can be misinterpreted by a reader. The analysis has found some evidence that cis-differences in TF binding may be one of the mechanisms for ancestry- associated differential CRE activity, not that it is a "likely" mechanism. Also, the "rather than differences in TF expression levels in trans" is not something that has been tested here.

We appreciate these comments highlighting areas where a reader could misinterpret our results. We have removed the phrase about TF expression levels, recognizing that we did not test for any association between TF expression levels and CRE activity, and have rephrased *cis* differences in TF binding as a “possible” rather than “likely” mechanism behind ancestry-associated differential CRE activity (line 328-329):

“Having established ancestry-dependent *cis* differences in TF binding as a possible mechanism for ancestry-associated differential CRE activity…”

The purport of the paragraph from line 321 to 340 is unclear. For instance, while the finding that "JunD, NF-κB, PU.1, and Oct1 bQTL have higher FST in diff-ATAC than in non-diff-ATAC enhancers" is syntactically clear, it is hard for the reader to judge what to make of it. Some of the other tests do not cross significance levels so no conclusion can be drawn.

We thank the reviewer for highlighting the lack of clarity around the conclusion to be drawn from this paragraph. We have further emphasized the conclusion of this analysis as the possibility that selection has acted on the binding of these TFs specifically in differential activity CREs (lines 330-335):

“We found that JunD, NF-κB, PU.1, and Oct1 bQTL have higher *FST* in diff-ATAC than in non- diff-ATAC enhancers (Wilcoxon P = 2.4 x 10^-9^, 5.0 x 10^-4^, 2.2 x 10^-4^, and 6.0 x 10^-4^, respectively), consistent with differential binding of these TFs as drivers of differential enhancer activity, as well as the possibility that their binding specifically in differential activity CREs has been subject to selection.”

We acknowledge that this association could be explained by genetic drift at the start of the following paragraph, but we would not have proceeded with our directional selection analysis without this finding.

Regarding claims about promoter, e.g., "enhancers rather than promoters display the strongest signs of selection associated with sites of differential transcription factor binding.", could the insignificant p-values in tests with promoters arise because differential promoter activity is harder to detect (less correlated) with differential ATAC-seq? If the detection methodology (diff-ATAC) is biased against detecting promoters for some reason, then the claims made here regarding differences in enhancer/promoter biology are not conclusive.

We appreciate the reviewer’s insightful comment into how methodology-dependent differences in ability to detect differential activity in enhancers versus promoters could affect our comparison of evidence for selection in each. Given our finding of differential enhancer enrichments for bQTL and lack thereof in differential promoters across both ATAC- and ChIP- defined differential CREs, this bias would need to exist across two independent methodologies to affect our results. We see similar proportions of enhancers and promoters defined as differential using ATAC and ChIP scores (20% and 18%, and 10% and 9%, respectively, for ATAC and ChIP, respectively) indicating similar levels of differential signal present in each across both methods. Moreover, given promoters displayed higher coverage than enhancers across both methods (see response to previous comment), if anything, we were more powered to detect differences in regulatory activity at promoters. We are not aware of any evidence suggesting ATAC-seq is better at detecting enhancer activity than at detecting promoter activity. To address this directly in the main text, we have included the percentages reported above in the paragraph in the Discussion section addressing this finding (lines 433-435):

“Moreover, we find similar proportions of diff-ATAC and diff-ChIP enhancers versus promoters (20% versus 18%, and 10% versus 9%, respectively) indicating similar levels of differential signal present in each across both methods.”

Minor comment:

In the definition of "ChIP-score_E,G", is it true that the \sqrt{H_G} term cancels out in the numerator and denominator, leaving the formula as one proportional to \sqrt{H_E}? What is the rationale for this relationship?

We thank the reviewer for highlighting that the fraction in our ChIP score definition is not expressed in the simplest form. We kept the *HG* term in both the ChIP and HiC score definitions for the reader’s convenience to compare these definitions with the ABC score definition to follow the logic of how we decomposed these scores and broke HiChIP down into its components. The promoter component cancelling out in the ChIP score makes regulatory activities more comparable between target genes and allows for comparison of these scores to ATAC scores (which don’t include target gene promoter activity).

# Reviewer #2:

In this manuscript, Pettie et al. examined the potential impact of ancestry-specific selection acting on chromatin activity, TF binding, and chromosomal looping. The authors build a strong case regarding the evolutionary and functional importance of regulatory DNA, and topics covered in their manuscript lie within the scope of Genome Biology. Although the text is a little dense in parts, the quality of the writing in this manuscript is acceptable. Similarly, the figures are serviceable. I liked the idea of looking at the level of chromatin to identify potential targets of adaptation that may have been missed by traditional DNA-based scans. Interestingly, the authors observe different signatures with respect to ancestry for ATAC-seq, ChiP, and HiC data. The innovation of this study and it's potential impact make it a good fit for a journal with an impact factor like that of Genome Biology. However there are three key technical and methodological issues thatdampen my enthusiasm for this manuscript.

First, all of the experiments used lymphoblastic cell lines (LCLs). How robust are the primary findings of this paper to the use of LCLs? Do ancestry differences in ATAC, ChIP, and HiC scores occur in other types of cells? I understand the difficulties re: obtaining other types of cells from ancestrally diverse populations. However, the authors need to build a stronger case that their results are robust to the choice of cell lines - especially since they are making the provocative claim that differential gene regulation across ancestries is modulated by chromatin activity. For example, how similar are ATAC,ChIP, and HiC scores for LCLs and whole blood of individuals who have the same ancestry? Note that buttressing this point need not require a complete set of additional experiments. Expansion of the text and inclusion of multiple supportive citations might end up being sufficient.

We thank the reviewer for highlighting the limitations imposed by our use of LCLs. To our knowledge our study is the first to enable explicit examination of ancestry-associated differential activity of regulatory elements in human cell lines or tissues, so we cannot directly answer the question of how ATAC/ChIP/HiC scores in other tissues would differ by ancestry. However, since ATAC-seq peaks and HiChIP loops have been shown to be highly cell type-specific, we would generally not expect the same peaks/loops to be shared across cell types. To address this point, in the Discussion we highlight the limitations of examining only LCLs (lines 447-450):

“…our study is limited by any changes to genome architecture introduced by Epstein-Barr virus in transforming B cells into LCLs that further mask the effects of any selection that has acted on B cells or even more relevant cell types…”

We also note that for the molecular traits that have been examined across human ancestries in both LCLs and other tissues, such as gene expression levels and DNA methylation, the population-specificity initially seen in LCLs has later been found to be shared in whole blood and other tissues (e.g., comparing gene expression in LCLs [2] vs PBMCs, and DNA methylation in LCLs [3] vs PBMCs).

Second, data were quite noisy across replicate pairs of experiments. Given that no less than 4 out of 16 key datapoints (2xCEU and 1xESN and 1xFIN) appear to be outliers, additional replicates are required. If strong claims about differences across ancestries are to be made it is imperative that that the results within each ancestry show greater consistency.

We appreciate the reviewer’s concern about reproducibility. ESN rep1 and FIN rep1 had the lowest numbers of HiChIP valid *cis* interaction pairs among all samples, which likely corresponds to low coverage regions driving their outlier status. Since these samples are not outliers and cluster more closely with their corresponding rep2s for the score types that yield our key findings (ATAC and ChIP), we are confident that ancestry-associated differences for these score types (which show clear separation by ancestry in our clustering analyses) are not due to spurious differences such as those that could be seen between replicates. We have adjusted the main text to provide more clarity around the HiC score outlier status of ESN and FIN rep1 and why we retained these samples for diff-score analysis (lines 192-195):

“Although FIN rep1 and ESN rep1 are also outliers for HiC scores, and thus also for ABC scores (Fig. S9-10, Additional file 1), this is likely driven by low coverage CREs since these are the two samples with the lowest number of valid HiChIP *cis* interaction pairs (see Fig. S1, Additional file 1).”

By contrast, both CEU replicates were outliers among ChIP scores. Since this score type is integral to our downstream findings and the CEU population represented 25% of our EUR samples, we removed these samples from our analysis to ensure our results were not driven by cell line age (see “*Outlier removal”* section in the Supplemental text of Additional file 1).

Third, the tests of population differentiation and natural selection can be enhanced. For example, Fst statistics are know to be lower for rare alleles that are due to recent mutations (think of a comparison between a population that has an allele frequency of 0 vs. an allele frequency of 0.001). Are the sets of SNPs in Figure 4A (non-diff vs. diff) and Figure 4B (promoter vs. enhancer) frequency-matched? If not, some sort of frequency binning (by allele age or global allele frequencies in the 1KGP) might be advisable. To strengthen the claims that natural selection contributes to differentiation between ancestries it would be good to examine other types of selection statistics, e.g., iHS or any of the statistics included in Abrahim et al. 2023 (https://doi.org/10.1371/journal.pgen.1010494).

We thank the reviewer for this insight into how allele frequency could be a confounding variable in our *FST* analysis and the potential to strengthen our claims around the contribution of selection to CRE activity differences between ancestries. To address this, we have performed an additional allele frequency binning analysis detailed in the main text and methods and visualized in new supplemental figures (S20-21) that shows our results are not driven by allele frequency differences between the tested CRE types (Results lines 347-355, Methods lines 828-831):

“Since *FST* can be correlated with allele frequency (*i.e.,* rare alleles introduced by recent mutation have low *FST*), we sought to assess whether higher *FST* for diff-enhancer bQTL was driven by differences in allele frequencies between CRE types. Performing the same tests in each of ten allele frequency decile bins, we find more enhancer bins than promoter bins with mean *FST* greater in diff- versus non-diff CREs (see Fig. S20-21, Additional file 1). Additionally, although binning reduces the power of each test, more of these bins have nominally significant differences in *FST* between diff- and non-diff enhancers. These results suggest that greater allele frequency divergence in differential activity enhancers is not dependent on allele frequency differences between the tested CRE types.”

“To control for possible allele frequency differences in our diff- versus non-diff CRE bQTL *FST* Wilcoxon tests, we took the combined set of diff- and non-diff CRE bQTL in each test, split them by mean allele frequency across AFR and EUR populations into 10 decile bins, and performed separate tests within each of these bins.”

We also performed the same tests using iHS scores from Johnson and Voight (2018) and observe no significant differences (see figure and methods included below); however, we do not necessarily expect signatures of within population selection such as iHS to be enriched in CREs that are differentially active between ancestries. Moreover, since iHS (and related tests such as XP-EHH) detects long haplotypes that have quickly risen in frequency, it lacks the SNP-level resolution required for the tests we’ve performed here. Most of the other selection statistics reported in Abrahim et al. (2023) do not estimate signatures of the recent positive selection we hypothesize has contributed to differential enhancer activity, so we do not expect them to be differential. We hope the reviewer understands these limitations of our tests and appreciate the helpful paper reference.

**iHS in diff- vs non-diff CREs. a)** iHS values [4] of TF bQTL and H3K4me3 QTL are shown as boxplots for diff- and non-diff CREs of each displayed score type separated by if the QTL is in an enhancer (top) or promoter (bottom) analogous to Fig. 4a. iHS for all populations were obtained from Johnson and Voight (2019) [4] and overlapped with bQTL in our CREs. For Wilcoxon tests analogous to those in our *FST* analysis, we used the maximum iHS observed across all populations.

Other technical details of this study seem to be in well-thought-out. The choice of a pooled study design to examine four African (ESN, GWD, LWK, and LWK) and four European (CEU, FIN, IBS, and TSI) ancestry populations is acceptable. Similarly, the additional methodological choice to resize ATAC-seq peaks to 500bp and retaining the top 150k peaks by read count seems reasonable. The methods used to reduce allelic mapping bias of H3K27ac HiChIP.

We thank the reviewer for this positive feedback indicating their agreement with the other methodological approaches taken in our study.

An additional consideration involves the recently published document by the National Academies of Science, Engineering and Medicine (Using Population Descriptors in Genetics and Genomics Research - A New Framework for an Evolving Field). While I personally disagree with Conclusion 5-4 in the NASEM document: "Given that underlying cellular and physiological mechanisms are expected to be universal among humans, the default practice in such studies should be to not use any descent-associated population descriptors," Pettie et al. should be cognizant that their manuscript is likely to receive intense critical attention. That said, as illustrated by Lea et al. 2022, Randolph et al. 2021, and Pettie et al. (GBIO-D-22-01655)… differential gene expression and chromatin can indeed differ across ancestries.

We appreciate and share the reviewer’s concern around possible interpretations and criticisms of our ancestry-associated differential chromatin findings. We are confident that our careful and conservative interpretation of our findings reduces the likelihood of critical responses and invalid interpretations. Moreover, Lea et al. 2022, Randolph et al. 2021, and other studies in human genomics that have reported ancestry-associated differences have to our knowledge not received such criticism. We have added a sentence to the Discussion to highlight that any ancestry- associated differences in chromatin and/or gene expression reported in our study or elsewhere do not imply differences in underlying cellular and physiological mechanisms (lines 445-447):

“Importantly, any ancestry-associated differences that may exist in the regulation of these pathways as a result of selection or drift do not imply differences in underlying cellular and physiological mechanisms.”

Additional comments:

I appreciated the honesty of the authors regarding the limitation of some of their marginally significant results (e.g., the impact of promoter or enhancer activity on ancestry differential expression).

We appreciate the reviewer’s acknowledgement of our conservative interpretation of findings.

Page 6, line 118: This is an interesting idea, but wouldn't selecting acting on the level of chromatin activity also leave some sort of signature in terms of DNA? The authors are encouraged to expand upon this section of the introduction. What I'm thinking about here are situations where putative signatures of selection on the level of chromatin activity and the level of DNA sequences are incongruent. How to interpret such signals? Note that the expansion of text related to this topic may be better suited for a discussion point.

We thank the reviewer for this careful consideration of the intuition behind our approach to look for selection at the level of chromatin. We acknowledge that selection acting at the level of chromatin will also leave a signature at the DNA level, but given that chromatin activity predicts TF binding better than DNA sequence does [5], as we highlight in the Introduction, we reasoned that selection may be easier to detect with the former. Sequence level changes often do not correspond to changes in regulatory function and gene expression, especially when regulatory activity is buffered by the binding of multiple TFs. In the converse incongruent case of differential regulatory activity in a region without genetic variants showing signatures of selection, the causal variant likely regulates the region’s activity more distally/in *trans*. We have added to the following text to the Introduction (lines 121-124) to emphasize this:

“This can lead to cases where sequence-level changes, even those disrupting TF binding sites, do not correspond to changes in regulatory function and gene expression when regulatory activity is buffered by the binding of multiple TFs.”

Page 9, line 192: I'm not a fan of removing both CEU replicates, as these constitute two out of 16 total data points.

We agree that the removal of CEU is not ideal. We originally included both CEU replicates in the analysis, which led to much stronger and more significant results, especially with respect to signals of selection. However, since both CEU replicates were outliers (perhaps due to their much older age, dating back to the 1970s), we chose the conservative approach of removing them.

Some figure legends are overly long (e.g., Figure 1 and Figure 3). The authors are encouraged to put some of these details in the main text or to split figures into a smaller number of panels.

We appreciate the reviewer pointing out these verbose figure legends and have moved some of these legends’ text to the main text as suggested and made additional edits for brevity (lines 180- 186):

“…ATAC scores represent the chromatin accessibility at the enhancer (Fig. 1b, teal gradients). ChIP scores estimate the enhancer-gene pair’s collective H3K27ac signal as the geometric mean of total HiChIP signal at the enhancer and gene promoter (also known as the vanilla coverage square root (VC-sqrt)) (Fig. 1b purple gradients). HiC scores estimate the contact frequency of the enhancer and promoter independent of H3K27ac levels by dividing the HiChIP signal from read pairs specifically connecting the enhancer and promoter (Fig. 1b, magenta gradient) by the VC-sqrt (see Methods).”

Page 18 (Figure 1): Something is strange in Figure 1C. Why do the ESN replicate pairs and the FIN replicate pairs not cluster together in the ABC and HiC sub-panels?

We appreciate the reviewer pointing out these outliers. These two replicates have the lowest numbers of valid cis interaction pairs in our HiChIP data (Fig. S1, middle), which leads to some E-G pairs with sparse HiC contact coverage (few reads explicitly linking that CRE to that target gene) that quantile and score normalization cannot fully correct for. This drives their outlier status in the HiC and ABC (since ABC incorporates the HiC component of HiChIP) sub-panels, but not the ChIP sub-panel because HiChIP scores are derived from the sum of valid cis interaction pairs at E and G (not just the pairs explicitly linking the two). We have adjusted the main text to provide more clarity around the HiC score outlier status of ESN and FIN rep1 and why we retained these samples for diff-score analysis (lines 192-195):

“Although FIN rep1 and ESN rep1 are also outliers for HiC scores, and thus also for ABC scores (Fig. S9-10, Additional file 1), this is likely driven by low coverage CREs since these are the two samples with the lowest number of valid HiChIP *cis* interaction pairs (see Fig. S1, Additional file 1).”

Page 20 (Figure 2). I'm not a big fan of the schematics shown in Figure 2 (the oddly shaped quadrants are a bit non-intuitive).

The main text does a much better describing the approach the authors took. That said, graphical abstracts of methods can be useful. The authors are encouraged to find alternative ways of representing the approaches described in panel C of this figure.

We thank the reviewer for highlighting the difficulty readers may have interpreting Fig. 2C. We tested multiple representations of our directionality test and found this to be the best (among our audiences). The gray gradient callout is meant to highlight that the oblong shape comes from the overlap portion of the two circles depicted in (a). The legend directs the reader to Fig. S13, where this derivation is more clear, but we found this illustration overly complex for a main text figure.

Page 23 (Figure 4). P-values for all comparisons should be shown

We thank the reviewer for pointing out that we only displayed nominally significant P-values. We have updated the figure to display all P-values for visual and informational completeness.

Page 31, line 783: It might be good to provide additional justification (a citation perhaps) regarding the denominator in the GO Analysis equation that was use to rank genes. Even though it seems appropriate, it would be good to state why this particular equation was used.

We appreciate the reviewer drawing our attention to this missing citation. We have named the metric in the methods and provided a citation where it was shown to have a low estimated false positive rate (lines 811-812):

“…genes ranked by the value of their most differential CRE according to the following T-test statistic [6]…”

References

1. Fulco CP, Nasser J, Jones TR, Munson G, Bergman DT, Subramanian V, et al. Activity- by-contact model of enhancer–promoter regulation from thousands of CRISPR perturbations. Vol. 51, Nature Genetics. Nature Research; 2019. p. 1664–9.
2. Lappalainen T, Sammeth M, Friedländer MR, ‘t Hoen PAC, Monlong J, Rivas MA, et al. Transcriptome and genome sequencing uncovers functional variation in humans. Nature. 2013 Sep 15;501(7468):506–11.
3. Fraser HB, Lam L, Neumann S, Kobor MS. Population-specificity of human DNA methylation. Genome Biology, 13: R8 (2012).
4. Johnson KE, Voight BF. Patterns of shared signatures of recent positive selection across human populations. Nat Ecol Evol 2018 24. 2018 Feb 19;2(4):713–20.
5. Peng PC, Khoueiry P, Girardot C, Reddington JP, Garfield DA, Furlong EEM, et al. The Role of Chromatin Accessibility in cis-Regulatory Evolution. Zufall R, editor. Genome Biol Evol. 2019 Jul 1;11(7):1813–28.
6. Zyla J, Marczyk M, Weiner J, Polanska J. Ranking metrics in gene set enrichment analysis: do they matter? BMC Bioinformatics. 2017 Dec 12;18(1):256.

**Second round of review**

**Reviewer 2**

Once again, this well written manuscript by Pettie et al. examines whether there is evidence of ancestry-specific natural selection on chromatin activity, transcription factor binding, and chromosomal activity. Despite the limitations of their dataset, the authors have done an admirable job of blending population genetic analyses with chromatin activity data from lymphoblastoid cells. Overall, the authors have done a reasonable job of responding to reviewer comments (including my own). Changes made to the text have resulted in an improved, more comprehensive manuscript. However, there is one key fix that needs to be made to warrant publication in Genome Biology (dealing with outlier samples).

Although I am generally satisfied with this revised manuscript, there is still a small chance that the continental ancestry-level differences that were observed could due to some other factor (as only a small number of samples are described in Figure 1C. In their response to reviewer comments the authors bring up the good point that both outlier CEU replicates were older samples (dating back to the 1970s). What are the ages of all of the other samples? Is there any known reason why the FIN rep1 and ESN rep1 samples were outliers for ABC and HiC? For the sake of completeness the authors are highly encouraged to included a supplemental table listing additional features/metadata related to all 16 of the samples. The idea here is to rule out batch effects or other possible causes of differences in chromatin activity between AFR and EUR individuals (like differences in sample preparation or ages of LCL cells).


Additional notes:

I am satisfied with the author's response to my point about the limitations of LCLs, including the revisions made to the discussion section.

The authors have done a good job of enhancing their tests of natural selection. I am glad that additional figures (S20-S21) and text now highlight that their results are not driven by allele frequencies between tested CRE types. Although the iHS tests ended up having negative results (lack of major differences in iHS between non-diff and diff-CRES), for completeness it would be good to include the QTL type x QTL iHS plot as an additional supplemental figure.

Lines 260-264: Please add additional statistical details in these sentences. What were the p-values and tests that were applied? How much did DE genes over lap more than expected by chance?

**Authors’ response to reviewers**

**Reviewer #2:**

Once again, this well written manuscript by Pettie et al. examines whether there is evidence of ancestry-specific natural selection on chromatin activity, transcription factor binding, and chromosomal activity. Despite the limitations of their dataset, the authors have done an admirable job of blending population genetic analyses with chromatin activity data from lymphoblastoid cells. Overall, the authors have done a reasonable job of responding to reviewer comments (including my own). Changes made to the text have resulted in an improved, more comprehensive manuscript. However, there is one key fix that needs to be made to warrant publication in Genome Biology (dealing with outlier samples).

We thank the reviewer for this this kind validation of our efforts to improve transparency and make the manuscript more reader-friendly.

Although I am generally satisfied with this revised manuscript, there is still a small chance that the continental ancestry-level differences that were observed could due to some other factor (as only a small number of samples are described in Figure 1C. In their response to reviewer comments the authors bring up the good point that both outlier CEU replicates were older samples (dating back to the 1970s). What are the ages of all of the other samples? Is there any known reason why the FIN rep1 and ESN rep1 samples were outliers for ABC and HiC? For the sake of completeness the authors are highly encouraged to included a supplemental table listing additional features/metadata related to all 16 of the samples. The idea here is to rule out batch effects or other possible causes of differences in chromatin activity between AFR and EUR individuals (like differences in sample preparation or ages of LCL cells).

We appreciate the reviewer’s attention and concern around these outliers contributing to differential signal. The reason behind ESN rep1 and FIN rep1’s outlier status for HiC and ABC scores is that these replicates had the lowest numbers of HiChIP valid *cis* interaction pairs among all samples. We have edited and expanded the main text to emphasize this explanation and assuage concern about batch effects or other confounders driving downstream results (lines 192-199):

“Although FIN rep1 and ESN rep1 are also outliers for HiC scores, and thus also for ABC scores (Fig. S9-10, Additional file 1), this is likely driven by low coverage HiC contacts since these are the two samples with the lowest number of valid HiChIP *cis* interaction pairs (see Fig. S1, Additional file 1; Additional file 2). For ChIP scores, which quantify the total H3K27ac signal at a CRE (not only that contributed by reads explicitly defining an E-G pair, as in HiC and ABC scores where the aforementioned low coverage effects manifest), these replicates are not outliers, so it is unlikely that coverage or batch effects contribute to any signal differences in this chromatin activity metric.”

While neither Coriell Institute nor the 1000 Genomes Project provide information on individual cell line age or other metadata beyond sex, we draw the reader’s attention to Additional file 2, which contains the HiChIP read numbers used to generate Fig. S1.

Additional notes:

I am satisfied with the author's response to my point about the limitations of LCLs, including the revisions made to the discussion section.

We appreciate the reviewer’s acknowledgment of our efforts to highlight the limitations of our study.

The authors have done a good job of enhancing their tests of natural selection. I am glad that additional figures (S20-S21) and text now highlight that their results are not driven by allele frequencies between tested CRE types. Although the iHS tests ended up having negative results (lack of major differences in iHS between non-diff and diff-CRES), for completeness it would be good to include the QTL type  x QTL iHS plot as an additional supplemental figure.

We thank the reviewer for suggesting these analyses to improve the robustness and comprehensiveness of our results. We agree that although the iHS test results could not reject the null hypothesis, it is useful to include them for completeness. We have inserted the relevant plot as our new Fig. S19 and added the following section to our Supplemental text in Additional file 1:

“*iHS analysis*

To assess evidence for selection with statistics beyond *F_ST_*, we performed tests analogous to those in Fig. 4a using iHS scores from Johnson and Voight (2018) and observe no significant differences (Fig. S19, see Methods); however, we do not expect signatures of within population selection such as iHS to necessarily be enriched in CREs that are differentially active between ancestries. Moreover, since iHS detects long haplotypes that have quickly risen in frequency, it lacks the SNP-level resolution required for the CRE-level tests in this study.”

Lines 260-264: Please add additional statistical details in these sentences. What were the p-values and tests that were applied? How much did DE genes over lap more than expected by chance?"

We appreciate the reviewer’s attention to these statistical details and agree that they should be provided in the main text. We have provided odds ratio ranges and upper P-value bounds for the hypergeometric (*i.e.*, one-sided Fisher’s exact) tests described therein as follows (lines 264-271):

“We found that differential gene directionality matched more often than expected by chance in the same contexts in which differential activity and DE genes overlapped more often than expected by chance, as well as in three additional contexts for diff-ATAC (hypergeometric OR = 1.71-2.54, P < 3.3 x 10^-4^) and five for diff-ChIP (OR = 2.90-3.70, P < 4.8 x 10^-4^). Five PBMC contexts [40] were nominally enriched for diff-ChIP matching DE (OR = 1.63-2.12, P < 0.05), though not significantly after multiple test correction. This was in contrast to genes identified by diff-ATAC CREs, which were only nominally enriched in four PBMC contexts [40] at lower odds ratios (OR = 1.64-1.82, P < 0.038, Fig. 2d; see also Fig. S13d, Additional file 1).”
